# Analyzing the most frequent disease loci in targeted patient categories optimizes disease gene identification and test accuracy worldwide

**DOI:** 10.1186/s12967-014-0333-8

**Published:** 2015-01-21

**Authors:** Roger V Lebo, Vijay S Tonk

**Affiliations:** Department of Pathology and Laboratory Medicine, Akron Children’s Hospital, One Perkins Square, Akron, OH 44308-1062 USA; Northeast Ohio Medical University, Rootstown, OH USA; Department of Pediatrics, Texas Tech University Health Science Center, 4th Street 3601, Lubbock, TX 79416 USA; Texas Tech University Health Sciences Center, El Paso, TX USA

**Keywords:** Most frequent diseases, Targeted patient categories, Disease identification, Test accuracy

## Abstract

**Background:**

Our genomewide studies support targeted testing the most frequent genetic diseases by patient category: (1) pregnant patients, (2) at-risk conceptuses, (3) affected children, and (4) abnormal adults. This approach not only identifies most reported disease causing sequences accurately, but also minimizes incorrectly identified additional disease causing loci.

**Methods:**

Diseases were grouped in descending order of occurrence from four data sets: (1) GeneTests 534 listed population prevalences, (2) 4129 high risk prenatal karyotypes, (3) 1265 affected patient microarrays, and (4) reanalysis of 25,452 asymptomatic patient results screened prenatally for 108 genetic diseases. These most frequent diseases are categorized by transmission: (A) autosomal recessive, (B) X-linked, (C) autosomal dominant, (D) microscopic chromosome rearrangements, (E) submicroscopic copy number changes, and (F) frequent ethnic diseases.

**Results:**

Among affected and carrier patients worldwide, most reported mutant genes would be identified correctly according to one of four patient categories from at-risk couples with <64 tested genes to affected adults with 314 tested loci. *Three clinically reported patient series* confirmed this approach. *First, only 54 targeted chromosomal sites* would have detected *all* 938 microscopically visible *unbalanced* karyotypes among 4129 karyotyped POC, CVS, and amniocentesis samples. *Second*, *37 of 48 reported aneuploid regions* were found among our 1265 clinical microarrays confirming the locations of *8 schizophrenia loci* and *20 aneuploidies altering intellectual ability*, while also *identifying 9 of the most frequent deletion syndromes. Third,* testing *15 frequent genes* would have identified 124 couples with a 1 in 4 risk of a fetus with a recessive disease compared to the 127 couples identified by testing all 108 genes, while testing all mutations in 15 genes could have identified more couples.

**Conclusion:**

Testing the *most frequent disease causing abnormalities in 1 of 8 reported disease loci [~1 of 84 total genes]* will identify ***~****7 of 8 reported abnormal Caucasian newborn genotypes*. This would eliminate ~8 to 10 of ~10 Caucasian newborn gene sequences selected as abnormal that are actually normal variants identified when testing all ~2500 diseases looking for the remaining 1 of 8 disease causing genes. This approach enables more accurate testing within available laboratory and reimbursement resources.

**Electronic supplementary material:**

The online version of this article (doi:10.1186/s12967-014-0333-8) contains supplementary material, which is available to authorized users.

## Background

Targeted testing the most frequent listed disease causing sites comprising <0.3% to 1.5% [This study] of the ~22,000 individual gene locations causing ~2500 total reported diseases [1] will minimize the number of incorrectly identified abnormal gene sequences by excluding nearly all normally variant sequences. By minimizing interpretation time and confirmation of abnormal results, this will enableready identification of most disease causing genomic sequences to expedite patient testing.

All laboratory tests have limitations. Because positive genetic test results may provide the basis for clinical decisions on both patients and their relatives for many years, clinical laboratories continually strive to offer optimal tests that maintain the highest possible reported accuracy. Genomewide analysis is limited by genome complexity so that accurate interpretation of all test results can be challenging. For instance, genomewide microarray analysis of 1,800,000 sites for disease causing rearrangements also identifies 906,600 polymorphisms and 5,677 copy number variants [2] among the ~2,370,000 total listed copy number variants [3]. Genomewide next generation sequencing continues to identify reported genetic mutations with ever more accurate, rapid, less expensive platforms that can analyze a few selected genes up to all ~22,000 genes in the 6,000,000,000 basepair diploid genome. Yet the more sites tested, the more normal sites are identified as abnormal by available data analysis. Currently “the rate limiting factor in clinical genomewide testing is the numerous variant gene sequences that multiply the cost of interpreting the raw sequence about 10-fold” [4].

Dr. James Watson, a healthy senior scientist was among the first three individuals to have his entire genome sequenced [5,6]. Three computer programs found Dr. Watson’s genome includes (1) two homozygous variants in genes previously reported to cause Usher Syndrome 1b and Cockayne syndrome, both early childhood diseases [7], and (2) a breast cancer gene sequence originally interpreted to reflect a major mutation among his >80 described mutated alleles. Subsequent reinterpretation of his known breast cancer gene variant avoided further clinical intervention [6]. None of these three diseases are among the most frequent selected for routine genomewide testing (Additional file 1: Table S1, Additional file 2: Table S2, Additional file 3: Table S3, Table 1).

**Table 1:** **Total population frequencies (Additional file** **1** **: Table S1A, B, C, D3, E right) by selected tested patient categories (Additional file** **1** **: Table S1D1, D2, E2)**

***Note: These frequencies will be substantially higher for symtomatic patients***				
**Disease categories**	**Caucasian**		**Worldwide+**	
**Tested**	**(a) Affected**	**(b) Heterozygote**	**(a’) Affected**	**(b’) Heterozygote**
***1. COUPLES, Asymptomatic, at risk***		***~1/132***		***~1/174***
***(1A2b +1B2b) (ASYMPTOMATIC,***		***(~.76%)***		***(~.58%)***
***2. FETUSES, Abnormal***	***~1/11.0***		***(1/11.1)***	
***(1A1a +1 B1a +2A1 + 3A)***	***(~9.2%)***		***(~9.0%)***	
***3. NEWBORNS, Affected***	***~1/52***		***~1/54***	
***(1A1a + 1B1a + 1Ca + 2c + 3A)***	***~1.91%***		***~1.86%***	
***4. ADULTS(a), Affected***	***~1/37***			***~1/43***
***(1A1a,1B1a + 1Ca +1 Da +2A +3A)***	***~2.71%***		***~2.33%***	

*Testing for Abnormal Genotypes in Asymptomatic Adults and Symptomatic Fetuses, Newborns, Children, and Adults.*

*Frequencies for each disease category are listed in* Additional file 1: Table S1 *according to the frequency in the general population. Clinically affected patients tested for any age-appropriate category carry substantially greater frequencies of affected genotypes* (Additional file 2: Table S2 and Additional file 3: Table S3). *Age appropriate tests are anticipated to optimally identify specific diseases in affected patients according to patient category* (Table 4)*.*

Carrier screening for cystic fibrosis has been applied to millions of patients and their at-risk partners according to this current standard-of-care DNA test [8,9] reported as either positive or with a residual negative test risk [10,11]. Newborn Screening Programs in every state test for selected abnormal metabolite concentrations by mass spec while some states also test newborns for cystic fibrosis mutations to optimize follow up care. [Additional file 1: Table S1A, Column 2, NB tested in Ohio] Frequent disease gene mutation screening selected for Ashkenazi patients by ACMG [12] and completed on tens of thousands of Ashkenazi patients from New York to Tel Aviv [13] is attributed with substantially reducing the frequency of affected newborns with these ethnic diseases.

Platforms that sequence the exome and those that quantify the copy number of targeted sites [14–16] together enable genomewide analysis to identify (1) single nucleotide substitutions, (2) gene deletions revealed by FISH, and (3) unbalanced chromosome region copy number abnormalities identified by karyotypes and precisely delineated by microarrays. Standard karyotyping is still the preferred method for detecting balanced and complex rearrangements, as microarray and sequencing methods are being validated to detect balanced abnormalities ([17], Results). Additional modifications to DNA analysis platforms have enabled sequencing single nucleotide mutations by microarrays [18] while other computer programs count and list the number of copies of each selected sequenced locus to quantify abnormal genomic sites c.f. [19].

Targeting genomewide screening the most *frequent genetic diseases affecting the largest proportion* of patients worldwide with rapid analysis platforms will enable unambiguously identifying more abnormal genotypes in at-risk couples and affected conceptuses and patients with fewer confounding results [Tables 1 and 2]. Testing products of conception and fetuses for the most frequent chromosome aneuploidies identifies the largest proportion of abnormal conceptuses [Additional file 2: Table S2, Col 2,3,4]. Testing abnormal infants and children identifies a substantial proportion of patients with altered intellectual development caused by a frequently deleted or duplicated submicroscopic chromosome region (Additional file 3: Table S3). Simultaneously testing for the other most frequent dominant and recessive single gene disorders including those in appropriate ethnic populations [Additional file 1: Table S1F] can provide accurate results to large patient populations within medical, laboratory, and reimbursement resources [Table 1].

**Table 2:** **Most frequent disease gene categories tested in patients**

	**Autosomal/X-Linked recessive**	**Chromosome/Gene R**	**Autosomal dominant/Aneuploidy**	**Late onset disease**
1. Reproducing or selecting partner	X			
2. At-risk conceptus or fetus	X	X		
3. Affected newborn or minor	X	X	X	
4. Affected adult	X	X	X	X

Legend: Carrier screening includes asymptomatic patients selecting partners, planning to conceive, or pregnant, and partners of identified carriers. Prenatal testing includes products of conception and at-risk fetuses. Symptomatic newborns and minors can be tested for autosomal dominant disease loci to determine the cause of their abnormal phenotype. Adults could be tested for selected late-onset disease genes and males for Y-linked infertility.

## Methods

### Design of the study

This study tested the hypothesis that analyzing the most frequent genetic diseases selected from all reported diseases would identify the largest proportion of disease causing mutations to unambiguously define each positive testing patient’s genetic abnormality with very few incorrect test results. For instance, when testing 100,000 patients for the frequent autosomal recessive cystic fibrosis mutations with 99.9% test accuracy per gene, a positive carrier test would include 3445 correct answers and 103 incorrect answers (Table 3, top; Ref. [20]). In contrast, when testing the rare fumarase deficiency gene locus with the same test accuracy, a positive carrier test would not only identify 26 carriers correctly but also 100 noncarriers as carriers [Table 3, bottom]. Thus, a minimal frequency of ~1 in 100,000 affected individuals for each listed abnormality was arbitrarily selected in each population analyzed to minimize incorrect test results while maximizing the number of genetic abnormalities identified. Available patient studies with the largest summarized experience [1] were selected to compile the abnormal gene frequencies in populations.

**Table 3:** **Carrier test accuracies for frequent and rare autosomal recessive diseases** [20]

**Disease**	**Disease frequency**	98% Accurate* 100,000 tested	99.9% Accurate** 100,000 tested	**Carrier frequency**
Cystic Fibrosis	~1/3364	~3279 Correct	~3445 Correct	~1/29
		~2067 Incorrect	~103 Incorrect	
		(2000 + 67 = 2067)	(100 + 3.3 = 103.3)	
PKU	~1/10,000	~1960 Correct	~1998 Correct	~1/50
		~2040 Incorrect	~102 Incorrect	
		(200 + 40 = 2040)	(100 + 2 = 102)	
Arylsulfatase A	~1/100,000	~619 Correct	~632 Correct	~1/158
Deficiency		~2012 Incorrect	~101 Incorrect	
		(2000 + 12.4 = 2012.4)	(100 + .6 = 100.6)	
Fumarese	~1/60,000,000	~25 Correct	~26 Correct	~1/3873
Deficiency		~2000 Incorrect	~100 Incorrect	
		(2000 + .5 = 2000.5)	(100 + .026 = 100.026)	

*A 96% to 98% accurate cystic fibrosis result frequency was initially reported by CAP certified testing laboratories.

**A 99.5% accurate result frequency was estimated by one commercial microarray manufacturer.

These data illustrate the prudence of testing maternal and fetal samples together.

LEGEND: One first reason for targeting the most frequent genetic diseases is illustrated by the calculated differences between correct and incorrect test results for diseases with different frequencies given the same test accuracies. For instance, considerably higher test accuracies are observed when calculated for screening of the more frequent autosomal recessive diseases in unselected asymptomatic carriers. The proportion of incorrectly detected carriers increases substantially for rare autosomal recessive diseases like Fumarase deficiency.

Clinical test accuracy is optimized during laboratory validation according to College of American Pathology guidelines. An illustrative 98% test accuracy has been arbitrarily selected for comparison of a Standard of Care test based upon the 96% to 98% accurate cystic fibrosis results reported by CAP certified clinical laboratories initially screening for the 23 most common cystic fibrosis mutations. Given a test accuracy of 98% for cystic fibrosis would identify ~3279 cystic fibrosis carriers correctly and ~67 carriers and ~2000 noncarriers incorrectly among 100,000 people. The same test accuracy applied to the rare autosomal recessive fumarase deficiency with a frequency of ~1 in 60,000,000 would identify 25 of 26 fumarase deficiency carriers correctly but also identify 1 carrier and ~2000 noncarriers incorrectly.

DNA sequencing platforms themselves are anticipated to be substantially more accurate, *while entire test accuracy is also modified by sample collection, laboratory manipulation, and reporting.* An arbitrarily selected 99.9% accurate test would decrease the incorrectly identified noncarriers for each genetic disease from ~2000 to ~100 among 100,000 patients tested. At the same time the number of correctly detected cystic fibrosis carriers would increase by 66 to 3445. In contrast, the 26 true carriers of the rare fumarase deficiency with a frequency of 1 in 60,000,000 would be identified correctly among the 100 incorrectly identified carriers. Compare these to the calculated 99.9% accurate test results for autosomal recessive Arylsulfatase A deficiency with an affected frequency of 1 in 100,000 that would identify 632 carriers correctly along with 1 carrier and 100 noncarriers incorrectly.

The ~50-fold enriched frequency of most frequent deletions found among all patients submitted for microarray analysis (Additional file 3: Table S3B, top) illustrates the principle that testing clinically suspicious phenotypes substantially enhances the affected patient frequency among tested samples. Prior screening test results like hemoglobin electrophoresis for sickle cell anemia and the hemoglobinopathies will further enrich for abnormal patient samples submitted for DNA analysis.

### Study setting

The diseases listed in descending order of frequency were identified from: (1) GeneTests 534 listed disease prevalences affecting at least 1 in 100,000 people among the >2500 listed diseases, (2) our 4129 reported products of conception and prenatal karyotypes in Ohio, (3) our 1265 reported patient microarray results in Texas, and (4) reanalyzed results of 25,452 prenatally screened women and their at risk partners tested for 108 disease genes [16].

### Type of participants and materials

The abnormal clinically reported prenatal karyotypes were derived from 1,449 products of conception (POCs), 82 chorionic villus samples, and 2598 amniocenteses completed at Akron Children’s Hospital from 2002 to 2013 (4,129 total cases). For comparison, we added the substantially lower frequencies of our previously published abnormal results on 25,222 amniocenteses and 5,134 chorionic villus samplings with a substantially larger proportion of patients of advanced maternal age among those cases with abnormal ultrasounds completed by 1992 [21].

The 121 (9.6%) clinically reported abnormal microarray results from 1265 Texas’ (T) patients were submitted for phenotypic abnormalities unrelated to oncology. These 40 different identified submicroscopic deletions and duplications each spanning about ~2,000,000 basepairs were reported with references in the Agilent and/or BlueGnome databases (Additional file 3: Table S3A, Ref. [22,23]). The positive results at each genomic locus were listed initially according to the relative frequency of each abnormal site observed [Additional file 3: Table S3A, Col 4, Left]. The frequencies reported in another developmentally delayed population of 15,749 cases and 10,118 controls [24] were added on the right side of the affected column for comparison [Additional file 3: Table S3A, Col.4, Right, (K)]. Available reported abnormal copy number frequencies in the general population were added in the next column for comparison [Additional file 3: Table S3A, Col 5].

These data were segregated further according to diseases with published population frequencies at the top in the order of chromosomal location for ready comparison [Additional file 3: Table S3B, top]. The remaining list was further segregated and ordered according to the chromosomal location of frequent deletions and duplications in patients with altered intellectual development [Additional file 3: Table S3B, middle], followed by patients with other clinical abnormalities [Additional file 3: Table S3B, bottom].

### Analysis

All available 534 frequencies reported in the GeneReviews chapters under Prevalence were collected in 2011 and updated when a significant change was noted. This selected disease list is further organized by transmission category: (A) autosomal recessive, (B) X-linked, and (C) autosomal dominant [Additional file 1: Table S1A,B,C]. Then Y-linked and mitochondrial diseases were listed just prior to the frequent diseases in specific populations [Additional file 1: Table S1D,E,F].

Then the abnormal post conception karyotype categories were listed in order of frequency in products of conception [Additional file 2: Table S2A, Col 2]. These included (1) 1449 Products of Conception analyzed from 2002 to 2013 in Ohio, (2) the first 5,134 CVS samples tested in San Francisco [21] next to the 82 high risk samples karyotyped in Akron since 2002, (3) the first 21,288 amniocenteses karyotyped in San Francisco [21] next to the 2,598 karyotyped in Akron since 2002, and (3) 54,749 newborns karyotyped in Seattle [25].

General population frequencies used in these calculations include 50% of abnormal karyotyped products of conception [POC] in Ohio by 2013 and 0.6% of abnormal karyotypes in newborns in Seattle by 1986 (Additional file 2: Table S2B, Col 2,5). In contrast, for prenatally sampled higher risk fetuses, abnormal karyotypes reported in chorionic villous samplings [CVS] and amniocenteses completed more recently in Ohio were compared to those completed by 1992 in San Francisco (Ref. [21]; Additional file 2: Table S2A,B, Columns 3,4).

The most frequent 48 submicroscopic aneuploid loci [Additional file 3: Table S3A] and their observed and reported frequencies were compiled from several sources: *(1)* The clinically reported 40 submicroscopic deletion and duplication sites each spanning >400,000 basepairs found in 1265 patients [Additional file 3: Table S3A, Texas (T); this manuscript], *(2)* thirteen (13) of the sixteen (16) deletions with estimated general population frequencies of at least 1 in 100,000 selected from GeneTests [Additional file 3: Table S3B, Top], *(3)* an additional 23 chromosome regions reported to result in altered neurocognitive development when deleted and occasionally when duplicated [Additional file 3: Table S3B, Middle], and *(4)* the additional 12 clinically reported abnormalities identified among our 1265 constitutional microarrays [Additional file 3: Table S3B, Bottom]. The first 13 are primarily syndromic deletions that frequently result in developmental delay [Additional file 3: Table S3B, Top]. The next group is reported to result in intellectual delay with or without other abnormalities. When testing for all these 48 abnormalities in 1265 patients referred for microarrays, 10% were reported positive (T): ~5% in the first 13 loci [Additional file 3: Table S3B, Top] and ~5% in the remaining 35 loci [Additional file 3: Table S3B, Middle, Bottom].

## Results

### Selection of frequent diseases

The most frequent listed disease locations comprise the largest proportion *of* testable disease causing mutations worldwide. Estimated disease frequencies found in all tested categories were derived from our calculations based upon reported patient and general population data [Additional file 1: Table S1, Additional file 2: Table S2 and Additional file 3: Table S3]. The most frequent disease alleles for cystic fibrosis and the hemoglobinopathies are reported to have been selected by heterozygous advantage [26–28]. In contrast, the other most frequent autosomal recessive genetic disease genes have many unique alleles but none were reported to have sufficiently frequent mutations that comprise a major proportion of all mutations (Additional file 1: Table S1A; Ref. [29]). Thus determining whether a variant gene sequence is normal at a frequent disease gene site should include a comparison to all confirmed mutations.

Other frequent diseases in ethnic populations result from a limited founder pool with offspring who regularly select a partner from among the offspring of all the founders [Additional file 1: Table S1F]. These ethnic populations can be tested effectively by targeting the few most frequent mutations in the founders’ rare disease alleles. Initially a population disease frequency may be overestimated when sampled from a region with a higher carrier frequency. Disease frequencies in founder populations can also skew panethnic population frequencies when offspring migrate together to specific geographic regions like in the United States where more centrally located populations can more often trace their ancestors to citizens who arrived earlier in the nation’s history.

The most extensive available disease frequency estimates with the broadest geographical distributions were found on the GeneTests website in the GeneReviews chapters. This study considered all the >2500 listed disease genes [1] for inclusion in at-risk and affected patient test categories. The disease list selected was further organized by transmission category: (A) autosomal recessive, (B) X-linked, (C) autosomal dominant, (D) Y-linked, (E) mitochondrial, and (F) frequent diseases in specific populations [Additional file 1: Table S1A-F, Tables 1 and 4]. From the 534 listed and updated GeneTests frequencies, 125 diseases were selected that are each reported to affect at least 1 in 100,000 Caucasian individuals [Additional file 1: Table S1A,B,C,D] including 122 diseases reported to affect at least 1 in 100,000 people worldwide [Additional file 1: Table S1A,B,C,D, excluding #1, 2, 38] and 55 disease genes frequent in specific populations [Additional file 1: Table S1F].

**Table 4:** **Summary of disease frequencies in total population**

**Disease category**	**Caucasian**		**Worldwide(f)**	
	**(a)Affected**	**(b) Heterozygote**	**(a’) Affected**	**(b’) Heterozygote**
**A1. Autosomal**	**~1/668(a)**	**~2/5**	**~1/967**	**~1/3**
**Recessive**	**~ (0.15%)**	**(~40%)**	**(0.10%)**	**(~34%)**
***A2. Couples***		***~ 1/174***		***~ 1/255***
		***(~0.58%)***		***(~0.39%)***
**A3. Late Onset**				
**Parkinson (ww)**	**~1/120**	~1/3.9*	**~1/200**	**~1/7**
**Hemochromatosis (cau) ~ (0.83%)**	**(~26%)**	**(0.5%)**	**(~14%)**	
**B1. X-Linked**	**~1/1065**	**~1/546**	**~1/1065**	**~1/546**
	**~ (0.094%)**	**(~.18%)**	**~ (0.094%)**	**(~.18%)**
***B2*** **.** ***Couples***		***~1/546***		***~1/546***
***(Recessive)***			**(~.18%)**	**(~.18%)**
**C. Autosomal**	**~1/123**	**~1/123**	**~1/123**	**~1/123**
**Dominant**	**~(0.81%)**	**(~.81%)**	**~(0.81%)**	**(~.81%)**
**D1. Abn POC**	**~ 1/2**	**~1/2**	**~1/2**	**~1/2**
**Karyotype**	**~ (50%)**	**~ (50%)**	**~ (50%)**	**~ (50%)**
**Quantification (54)**	**~ (47.7%)**	**~ (47.7%)**	**~ (47.7%)**	**~ (47.7%)**
**D2. Current Abn Amnio**	**~ 1/13.8**	**~1/13.8**	**~1/13.8**	**~1/13.8**
**Karyotype**	**~ (7.2%)**	**~ (7.2%)**	**~ (7.2%)**	**~ (7.2%)**
**Quantification (54)**	**~ (6.1%)**	**~ (6.1%)**	**~ (6.1%)**	**~ (6.1%)**
**D3. Abnormal Newborn**	**~1/156**	**~1/156**	**~1/156**	**~1/156**
**Karyotype**	**~(0.64%)**	**~(0.64%)**	**~(0.64%)**	**~(0.64%)**
**Quantification (54)**	**~(0.59%)**	**~(0.59%)**	**~(0.59%)**	**~(0.59%)**
**E. Common**	**~** ***1/1097X 2(e)***	**~** ***1/1097X 2(e)***	**~** ***1/1097X 2(e)***	**~** ***1/1097X 2(e)***
**Deletions**	**~(0.18%)**	**(~.18%)**	**~(0.18%)**	**(~.18%)**
**F. Y-linked**	**~1/12,500**		**~1/12,500**	
**Hemizygote**	**~(0.008%)**		**~(0.008%)**	

(a) Without late onset hemochromatosis and Parkinson, with cystic fibrosis and α-1-antitrypsin in Caucasians.

(b) E1. First 5 abnormal karyotypes listed in Additional file 1: Table S1E detect 1/329 of 1/184 [.31% of .54%]. Other abnormalities may not be detected with targeted platform.

(c) E2. Other karyotypic abnormalities that may not be identified by a targeted platform. Lebo et al., 2002, lists additional 30 chromosome regions that would identify ~97% of all abnormalities if tested for copy number. Platforms with SNPs will identify copy number changes in any region in which these are found.

(d) Adult estimate excludes trisomy 13 and trisomy 18 from category (b) above.

WW = worldwide; Cau = Caucasian.

*Compare to Cystic fibrosis: [1/29]^2^ X [1/4] =1/3300]. Includes hemochromatosis.

(e) The frequency of the common deletions listed at the top of Additional file 1: Table S1E comprised 5% of the abnormalities identified by microarrays while the remaining 35 loci at the bottom comprised the other 5%. Thus the first population frequency was multipled by 2 to estimate the total frequency.

(f) Without common regional diseases.

Additional estimates of disease gene frequencies have been derived from our tested at-risk and abnormal patient samples [Additional file 2: Table S2, Col 2.3.4; Additional file 3: Table S3]. Initially the abnormal karyotype categories are listed according to decreasing frequency in clinically reported products of conception [Additional file 2: Table S2A]. These karyotype results were then reorganized according to estimated severity to facilitate comparison of the remaining viable karyotypic abnormalities as gestation progresses [Additional file 2: Table S2B]. Calculated general population disease frequencies include 50% of abnormal karyotyped products of conception [POC] and 0.64% of abnormal karyotypes in newborns [Additional file 2: Table S2B, Col 2,5, Bottom]. In contrast, for prenatally sampled fetuses tested by chorionic villus sampling [CVS], abnormal karyotypes were reported in 3.1% completed by 1992 in San Francisco [21] compared to 33% completed more recently in Ohio (Additional file 2: Table S2B, Col 3]). Note also the increased number of abnormal chromosome categories in the more recent sampled amniocenteses [Additional file 2: Table S2B, Col 4, Right, underlined).

Then the most frequent 40 aneuploid locations spanning >400,000 basepairs characterized by our microarrays were organized according to the frequencies of each abnormality among our 1265 tested patients [Additional file 3: Table S3A]. These frequencies among Texas’ patients (T) were subsequently reorganized according to chromosome location within 3 categories to facilitate comparison of (1) the 13 confirmed gene loci with estimated general population frequencies of at least 1 in 100,000, (Additional file 3: Table S3B, Top, Right Column, Ref. [1]) (2) the additional reported chromosome regions that result in altered neurocognitive development with or without other abnormalities ((K); Additional file 3: Table S3B, Col 4; Ref. [24]), and (3) the remaining 12 abnormal clinically reported loci. Nearly all frequent deletions listed at the top of Additional file 3: Table S3B also result in altered developmental delay. When testing for all these abnormalities, 10% of patients were reported positive for gene deletion or duplication in this Texas’ cohort.

**Figure 1:**
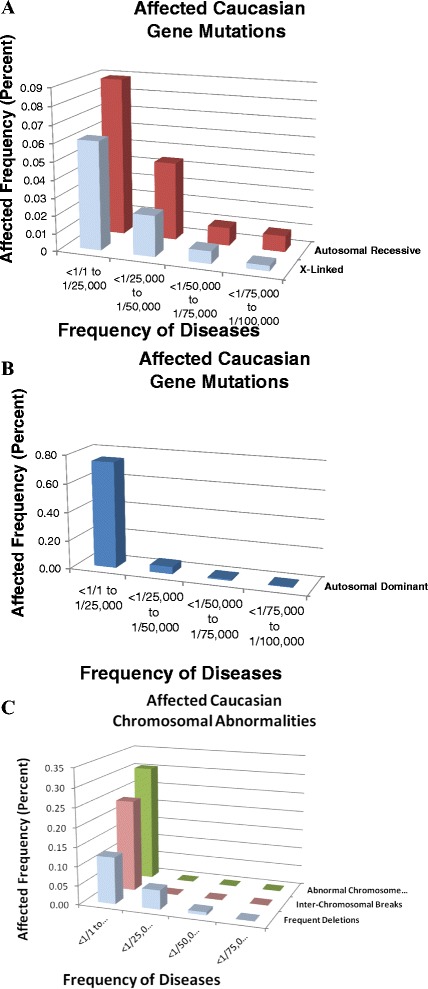
**Affected disease frequencies in four disease categories in caucasians (A,B,C).** The individual contributions of four disease frequency categories were graphed according to affected total frequencies (percent) for 6 disease categories of surviving patients in increments of 1 in 25,000. Note the frequencies of the first three categories were graphed with a frequency up to .09% for autosomal recessive, (Additional file 3: Table S3C), three categories were graphed on different scales with a frequency up to.30% for whole chromosome aneuploidy (Additional file 3: Table S3E), and autosomal dominant with a frequency up to .75%. 1. Among all the diseases with a frequency of at least 1 in 100,000, 86% of at-risk couples for an affected fetus with an autosomal recessive disease would be identified by testing only diseases with a frequency up to 1 in 50,000; 2. 91% of at-risk couples for an affected fetus with an X-linked disease would be identified by testing only diseases with a frequency up to 1 in 50,000; 3. 92% of the patients affected with an auatosomal dominant would be identified by testing only diseases with a frequency up to 1 in 25,000; and 98% with frequencies up to 1 in 50,000; and 4. All frequent duplications and chromosome abnormalities listed have frequencies exceeding 1 in 25,000. Given that most of these autosomal recessive disease genes have ~50 unique mutations with no particularly common mutations, [29], decreasing initial screening to diseases with at least 1 in 50,000 will not only substantially reduce the workload but will miss <1 patient per disease category in 2.5 years by a laboratory randomly screening 5,000 normal patients per year. These thresholds may need to be revised because the abnormal genomic frequencies of affected patients would be substantially greater.

The frequencies of six disease categories with available general population frequencies [Additional file 1: Table S1A,B,C; Additional file 2: Table S2, Newborn; Additional file 3: Table S3B,Top] were calculated and graphed for ready interpretation [Figure 1A,B,C]. Note that the largest proportion of reported disease frequencies is found in the first subcategory [1/1 to 1/25,000]. These proportions diminish rapidly as the affected patient frequency decreases in increments to [<1/75,000 to 1/100,000]. This consistent trend can be applied to optimally select disease inclusion frequency as ongoing test experience accumulates. These graphs demonstrate that any platform that tests the most frequent disease genes according to physician selected or peer reviewed disease categories will expedite reporting the largest proportion of clearly positive and negative test results.

*Altogether the first 227* [125 + 54 + 48; Additional file 1: Table S1, Col.1; Additional file 2: Table S2, Table 5; Additional file 3: Table S3A] *most frequent Caucasian diseases at 263 loci* [227 + 36; See Additional file 1: Table S1A,B,C,D, Column 4] *result from mutations involving ~1.2% of the ~22,000 human genes* [263/22,000 = 1.19%]*. This total is 224 diseases for worldwide patients* [122 + 54 + 48]. These values facilitated deriving the frequencies of at-risk asymptomatic couples carrying autosomal recessive [Table 4.A.2] and X-linked recessive diseases (Table 4.B.2) along with the dominant and Y-linked patient categories [Tables 1 and 4].

**Table 5:** **Genetic disease loci in critical chromosome regions**

**Chromosome band tested**	**Gene**	**Disease locus tested**	**Disease frequency**	**OMIM#**
1p36.3	MTHFR	Homocystinuria due to MTHFR		236250
		deficiency		607093
1q44	CIASI	FCAS Muckle-wells syndrome	N.A.	606416
		CINCA syndrome		
2p25	TPO	Thyroid peroxidase deficiency	N.A.	274500
2q37 N.A.	UGT1A1	Crigler-Najjar Syndrome, Type II Gilbert Syndrome	N.A	606785
3p25-p26	VHL	Von Hippel-Lindau Syndrome	N.A.	193300
3q27 or	TP63	Tumor protein P63	N.A.	603273
3q28	LPP	Lipoma-Preferred partner	N.A.	600700
4p16.3 or	FGFR3	Achondroplasia	1/20,000	100800
4p16.3	HD	Huntington Disease		143100
4p35	FSHMD1A	Facioscapulohumeral muscular dystrophy	1/250,000	158900
5p15.2-15.3	MSR	Methionine Synthase Reductase	N.A.	602569
6p25 or	FOXC1	Iridogoniodysgenesis	N.A.	601090
6p25-p24	F13A1	13coagulation enzyme	N.A.	134570
6q27	TBP	Spinocerebellar ataxia 17	N.A.	600075
7p22	MAD1L1	Somatic lymphoma	N.A.	602686
7q11.2	ELN	Williams Syndrome	1/10,000	194050
				130160
7q36	PRKAG2	Wolff-Parkinson-White Syndrome	N.A.	602743
8p23 or	MCPH1	Microcephaly, autosomal	N.A.	607117
8p22	LPL	recessive 1	1/10,000	238600
		Hyperlipoproteinemia I		
8q24.3	ZIP4	Acrodermatitis enteropathica	N.A.	607059
9p24.2	PDCD1	Mouse model develops lupus*	N.A.	605724
9q34.3	AGPAT2	Berardinelli-Seip	N.A.	603100
		Congenital Lipodystrophy 1		
10p15	GATA3	Hypoparathyroidism, sensorineural	N.A.	131320
10q26	AOT	Ornithine Aminotransferase Deficiency	N.A.	258870
11p15.5	CDKNC1	Beckwith-Wiedemann Syndrome	N.A.	600856
11q24	KCNJ1	Bartter Syndrome, Type 2	N.A.	600359
12p13.3	VWD	Von Willebrand Factor Deficiency	1/20,000	193400
12q24.2	TCF1	Diabetes Mellitus	high	142410
		Transcription Factor 1		
13q34	IRS2	Diabetes Mellitus Insulin receptor substrate		600797
14132.33	IGHM	Agammaglobulinemia	N.A.	147020
15q11.2	SNRPN #	Prader-Willi Syndrome	1/15,000	176270
	UBE3A #	Angelman Snydrome	1/15,000	601623
15q26.1	RECQL3	Bloom Syndrome	N.A.	606410
16p13.3	HBA1	Alpha Thalassemia	(C)	141800
				41850
16q24.3	FANCA	Fanconi Anemia	(D)	227650
17p13.3	LIS1	Miller-Dieker Syndrome	(E) 90% deletions	247200
17p11.2	PMP22	CMT1A/HNPP	1/5,000(F)	
			20% de novo	162500
17q25.3	HSS	Sanfilippo Mucopolysaccharidosis	(G)	605270
		Type IIIA		252900
18p11.3	TGIF	Holoprosencephaly	N.A.	602630
18q23	CYB5	Methemoglobinemia	N.A.	250790
19p13.3	ELA2	Cyclic Hematopoiesis	N.A.	130130
19q13.4	TNNT1	Nemaline myopathy	N.A.	191041
20p13	AVP	Diabetes Insipidus	N.A.	192340
		Neurohypophyseal		125700
		Arginine Vasopressin		
21q22.3	ITGB2	Leukocyte adhesion deficiency	N.A.	116920
				600065
22q11	DGCR	DiGeorge Syndrome	N.A.	188400
22q13.3	DIA1	Methemoglobinemia	N.A.	250800
		Diaphorase deficiency		
Xp22.32	STS	X-linked ichthyosis	1/5,000	308100
			Deletions:	
			90%	
Xp22.32-pter	SHOX	Short Stature Homeo Box	N.A.	604271
				312865
Xp21.2	DMD	Duchenne Muscular Dystrophy 65% deletions, 7 sites, 90%, 1/3 new mutations	1/4,000	310200
Xq28	SLC6A8	Creatine deficiency syndrome		300352
		X-linked		300036
Yp11.3	SRY	Sex-determining region Y		480000
		Godndal dysgenesis, XY type		
Yq11.2	USP9Y	Azoospermia		400005

Reproduced from Lebo et al. [30].

Frequent abnormal alleles in populations that are likely to select a partner from within their own ethnic group account for virtually all of the remaining homozygous autosomal recessive affected conceptuses. Altogether GeneTests reported 55 diseases that each affects at least 1 in 100,000 patients in specific ethnic populations [Additional file 1: Table S1F]. For the general U.S. population, the first five listed ethnicities each comprise at least 2% of the total U.S. population and together have 31 ethnic specific diseases that can be added readily to the general population screening test [Additional file 1: Table S1F, #1-31]. Most ethnic gene mutations require substantially less effort to test because these typically include very few frequent abnormal alleles.

The summary of these individual calculated affected and carrier disease frequencies are listed [Table 4] as well as the composite rates for Caucasian and Worldwide patient categories [Table 1]. These most frequent disease lists were constructed to facilitate physician-selected disease testing that could be ordered for four patient categories: (1) patients selecting a partner or reproducing, (2) at-risk conceptuses, (3) abnormal newborns and minors, and (4) affected adults [Tables 1 and 2].

Currently next generation platforms and their modifications can be used immediately to rapidly sequence the total exome or ~4800 disease related genetic sites for pregnant couples and affected newborns and adults. Analyzed genes can be selected from among the total results. Fetuses at a 1 in 4 risk of a known gene defect are tested for this risk first. Karyotypes are being ordered initially in Ohio for small invasive prenatal samples, very late gestation fetuses and 90% of refrigerated POCs that are cultured and karyotyped. Other facilities order microarrays [31] for sufficient CVS and amniocyte samples and up to 40% of POC samples that can not be cultured.Platforms that complete multiple test categories as well as the most important fetal test will continue to be developed and selected from the most reliable source as test platforms evolve and updated databases are constructed and maintained (Refs. [1,32]; Peer-reviewed publications).

#### Identifying at-risk couples

Testing asymptomatic patients prior to or during pregnancy can determine whether both partners carry the same recessive disease gene or the female partner carries an X-linked gene conferring a 1 in 4 risk of each conceptus being affected. Reported population frequencies would identify *40.2% of Caucasians as carriers of one of the 37 most frequent early onset autosomal recessive diseases* [Additional file 1: Table S1A; Table 4.A1.b]. Testing the other current partner will complete the goal of the screening test by identifying the 1 in 174 asymptomatic couples that both carry the same disease allele and have a 1 in 4 risk of an affected conceptus [Table 4.A.2.b]. Simultaneously the ~1 in 546 women [Table 4.B1.b] would be found who carry 1 of 21 most frequent X-linked recessive disease alleles with lower female carrier frequencies [Additional file 1: Table S1B]. Women who test positive for one of these genes have a 1 in 4 risk of an abnormal male fetus so that fetal testing would be offered without testing the partner. Taken together, ~1 in 132 (.76%) Caucasian couples are at a 1 in 4 risk for one of the first 61 frequent recessive diseases [Table 1.1.b, left] and 1/174 Worldwide [Table 1.1.b’ , right].

Compare targeted testing to the results of screening 23,453 asymptomatic patients that found *29.2% [24.0% one allele +5.2% more than one allele] Caucasian and ethnic carriers of one of 108 rare and frequent recessive diseases* [16]. Testing partners found 1 in 127 couples at a 1 in 4 risk for an affected fetus with 1 of 18 genetic diseases among the 108 tested [16]. Our further analysis found *124 of these 127 at-risk couples* in one of two populations: (1) *111 of 127 couples at-risk* for *1 of 8 frequent Caucasian diseases* (Additional file 1: Table S1A,B); and (2) *13 of 127 couples at-risk for 1 of 7 ethnic diseases* in couples with ancestors from the same subpopulation (Table two in Ref. [16]; Additional file 1: Table S1F, This mss.). *Only 3* couples were a risk for 1 of the 91 additional less frequent to rare tested diseases [16].

Subsequently our literature study of each of the 34 most frequent worldwide autosomal recessive genetic diseases [Additional file 1: Table S1A] found 28 to 652 reported disease causing mutations [29]. In contrast to cystic fibrosis and the hemoglobinopathies, the 34 worldwide diseases studied had no common mutant alleles so that heterozygous carrier advantage did not select for a few common mutations. *Taken together, these comparisons illustrate the efficacy of selecting the most frequent genetic diseases identified and testing for all confirmed disease causing mutations.*

Because differences in genetic disease severity or family history may modify the couple’s concern about a fetus affected with a reported disease [33], letters a, b, or c have been for more to less severe diseases [Additional file 1: Table S1A, 1–37] Discovering a 1 in 4 risk of an affected fetus for an autosomal recessive disease carried by both partners has modified mate selection. Discovering a 1 in 4 risk of an affected fetus for either an autosomal recessive or X-linked disease enables a couple to select other reproductive options prior to or during pregnancy or to optimize care after delivery. When testing each at-risk fetus, simultaneously testing parental samples including any new partners provides optimal controls to further confirm the fetal test result and maintain test accuracy. Reporting only abnormal prenatal results based only on previously reported disease causing genotypes substantially simplifies writing the most accurate reports, counseling, and a couple’s decision.

### Products of conception and prenatal screening

Akron Children’s product of conception [POC] protocol includes a pathologist’s examination, description, biopsy, histology, and submission of freshly biopsied chorionic villi for karyotyping. Chorionic villus biopsies are preferred to other fetal tissues because 90% of viable submitted fetal samples maintain cell viability when refrigerated up to 5 days prior to cell culture or DNA analysis compared to the poorer viability of other fetal tissues. Histologically prepared villi are analyzed for mole and partial mole morphology (Additional file 4: Table S4). The cytogenetics laboratory assures dissection of the villi to 90-95% purity for karyotyping and 98-100% purity for molecular analyses. After digestion and culturing, chorionic villus cells from 90% of submitted placental samples have been karyotyped. (Additional file 2: Table S2A). Following a preliminary report, polymorphic DNA identity testing confirms the fetal origin of the cultured samples prior to a final report. Of the remaining 10% of failed cultures, 7% are sufficiently intact to analyze interphase nuclei by FISH for chromosome 13, 16, 18, 21, 22, X, and Y aneuploidy comprising ~63% of all chromosome abnormalities in our karyotyped POCs. These 50% of cultures with abnormal reported karyotypes among all karyotyped POCs are listed in order of frequency for each category [Additional file 2: Table S2A, Col 2]. Given the 90% of samples karyotyped, these abnormal frequencies are interpreted to reflect typical POC population frequencies. ACOG recommends using microarrays for POC samples when 20%-50% of samples fail to grow in cuture and cannot be karyotyped [34]. Given 90% of our POCs are karyotyped according to our protocol, we have karyotyped all cultured POCs to identify tetraploidy from diploid genomes and balanced or complex categories of chromosome abnormalities currently missed by microarrays and genomewide sequencing.

Sampled at-risk fetuses have been karyotyped following CVS or amniocentesis to serve as the clinically standard test for microscopically visible chromosome abnormalities. For comparison we added the most recent 12 years of prenatal karyotypes in Ohio to our published 25 years of prenatal karyotypes in San Francisco from 1970 to 1995. This comparison found a 10.6-fold increase in more recent abnormal CVS karyotypes [3.1% to 33%] and a 3.2-fold increase in more recent abnormal amniocyte karyotypes (2.3% to 7.2%; Ref. [21]; Additional file 2: Table S2A, Col 3,4) These substantially higher abnormal frequencies in Akron’s more recent samples are consistent with those published recently by the American College of Obstetrics and Gynecology [34].

Subsequently these results were organized by severity to emphasize the evolution of surviving abnormal karyotypes as the time after conception increases [Additional file 2: Table S2B)]. These POC frequencies were compared to the reported frequencies in CVS, amniocentesis, and newborn karyotypes (Additional file 2: Table S2B, Col 2,3,4,5; Ref. [21]). Additional categories were observed in POC specimens: (1) triploid to aneuploid,(2) tetraploid to aneuploid, and (3) aneuploid to tetraploid [Additional file 2: Table S2B Col 2, Top, Underlined]. Additional categories were also observed in the more recent amniocyte samples over those previously published: (1) double aneuploidy, (2) isochromosomes, (3) complex abnormalities with two or more abnormal chromosome categories, (4) diploid to tetraploid, (5) trisomy 16 and 22, (6) monosomy 11 and 21, and (7) mosaic karyotypes [Additional file 2: Table S2B, Col 4, Underlined, Italicized].

The 54 selected sites reported to comprise the most frequent chromosome regions involved in abnormal chromosome copy number (Table 5, Reproduced from Ref. [30]; Derived from [32]) were found to have identified all the abnormal chromosome copy number regions identified in the listed abnormal POC, CVS, and amniocyte karyotypes in Akron [Additional file 2: Table S2B]. Because 2,500,000 site polymorphic microarrays only detect abnormal copy number, the frequency of abnormality detection would not have been increased by this assay, but the chromosome region spanned by the copy number change would have been delineated. These abnormal 54 aneuploid loci can be identified on any platform that (1) detects a sufficient number of the most informative adjacent single, di- and tetra-nucleotide polymorphisms and quantifies the relative and total number of times each of these sites were sequenced or (2) the relative number of targets at the aneuploid site compared to a normal diploid control region. Candidate platforms include rapid sequencers and microarrays. Following initial identification of an aneuploid chromosomal gene region on a 54 site test, the extent of the aneuploid gene region could be delineated readily by completing a typical polymorphic genomewide microarray or a ~4800 disease gene sequence.

*Targeting these 54 sites can readily serve as the next generation screening test of circulating placental DNA in maternal circulation [cfDNA] to enable genomewide aneuploid chromosome analysis.* These 54 sites comprising the most frequent microscopically visible aneuploid chromosome regions would include sufficiently large genomic targets to compare many polymorphic sites for small differences contributed by fetal DNA. The results provided by current and updated placental DNA screening tests are anticipated to continue to be more accurate than prior screening tests. Assuring that reported posterior test accuracy is reported correctly will enable the most confident initial and subsequent screening test utilization.

Alternative platforms and karyotyping should be compared carefully for different applications as tests evolve. For instance, among the ~50% of karyotypically abnormal POC samples, ~45% of these abnormal samples would have been detected by either a 54 site or a 2,500,000 site genomewide test platform provided the control DNA includes an intact Y chromosome [Additional file 2: Table S2B, not italicized]. The same platforms would have defined copy number changes in 5.7% of the 7.2% [(79%); (7.2%-1.54%)] abnormal amniocyte karyotypes and in ~25.7% of the 33% [(78%); (33%-7.3%)] current abnormal CVS karyotypes [Additional file 2: Table S2B].

Compare this to microarrays with 2,500,000 sites that precisely map unknown and previously detected unbalanced rearrangements and submicroscopic aneuploidy. These extensive microarrays are particularly useful in delineating the 6% of inherited cases with undetected submicroscopic deletions or duplications not detected in karyotypes of a conceptus with an apparently balanced chromosomal rearrangement inherited from a normal carrier parent [35]. In spite of the propensity of unequal chromosome recombinations among the potentially confounding ~2,370,000 copy number variants [3,36], balanced rearrangements are beginning to be detected by improved genomewide platforms and sophisticated computer analysis [17,37]. Karyotyping is the only means to detect diploid to tetraploid mosaicism and tetraploidy arising from diploid cells. As microarray, sequencing, and flow sorting methods for detecting balanced abnormalities are being validated in additional patients with substantial analytic resources,[17] standard karyotyping is still the preferred method for detecting balanced and complex rearrangements in a few viable cells.

Selecting the most optimal platform for any individual fetus will depend upon available ongoing test deveopment and available protocols through referral laboratories. At our location we karyotype all CVS samples because of the 33% found to have abnormal karyotypes. Currently we karyotype amniocytes for substantially abnormal ultrasounds and select the fetal samples to be sent for microarray analysis following following rapid FISH overnight. Our first sample designated for microarray testing was positive for trisomy 21 by Rapid FISH and subsequent karyotyping.

### Symptomatic newborns and children

Pediatricians following standard of care can decide to test symptomatic newborns and children for all frequent genetic disease categories that may define the abnormal phenotype. These disease categories include the autosomal dominant diseases resulting in ~1 in 123 affected newborns [Additional file 1: Table S1C, Table 4C] and the frequent deletions and duplications causing an estimated ~1 in 549 affected newborns [1/1097 X 2; Additional file 3: Table S3A,B, Table 4E]. Together these and all previously mentioned genetic disease categories [Table 1.3] result in ~1 in 54 [1.86%] affected newborns worldwide and 1 in 52 [1.91%] Caucasian newborns.

Calculated dominant disease prevalence reflects a disease frequency that includes many heterozygous abnormal genotypes [2pq] and very few homozygous affected patients [q2] in randomly mating populations with reproducing patients. Mutations involving 60 of these frequent autosomal dominant diseases [Additional file 1: Table S1C, Table 4C] include a substantial proportion that resulted from de novo mutations [2% to 95%]. As anticipated, these de novo autosomal dominant mutations are often more severe than autosomal dominant diseases inherited from reproducing adults. If an autosomal dominant disease gene were identified in an affected minor, parents could then be counseled and offered testing to determine whether the minor’s disease gene is de novo [including parental germ line and somatic mosaicism], or resulted from substantial anticipation, variable penetrance, or variable expressivity. Testing these autosomal dominant diseases requires a platform with the sensitivity to detect single nucleotide mutations.

The submicroscopic deletion and duplication category was addressed by analyzing the prior 5 years of abnormal microarray results that each spanned >400,000 basepairs in our Texas’ patient cohort. These samples were submitted in order of highest to lowest frequency by Neonatologists, Pediatric Neurologists, Geneticists, Developmentalists, and Pediatricians. The 9.6% abnormal results [121/1265] were organized into three categories [Additional file 3: Table S3B]: (1) 13 loci with frequent deletions with reported normal and abnormal population frequencies that nearly all involved altered neurocognitive development [Additional file 3: Table S3B, Top], (2) at least 23 additional reported loci related to altered neurocognitive development with reported frequencies in abnormal patients [Additional file 3: Table S3B, Middle], and (3) 12 additional clinically significant loci including three chromosomal abnormalities. [Additional file 3: Table S3B, Bottom] About 1/2 of the 123 total deletions and duplications spanned 9 of the first 13 recurrent deletion loci. The other ~1/2 of the cases in categories 2 and 3 included 8 recurrent schizophrenia loci [24,38].

A 52-fold enrichment was found between the 1 in 21 affected patients selected for microarray testing in Texas and an estimated 1 in 1097 patients predicted in the general population by estimated disease frequencies [Additional file 3: Table S3B, Top]. This ~50-fold enriched frequency of most frequent deletions found among all patients submitted for microarray analysis illustrates the principle that testing clinically suspicious phenotypes substantially enhances the affected patient frequency among tested samples [Additional file 2: Table S2 and Additional file 3: Table S3]. Although a chromosome abnormality was not suspected, 15 cases with trisomy 21, trisomy 18, or iso(12p) were identified by microarrays in our Texas cohort. Given the difficulty in identifying the exact genetic abnormality by the patient phenotype alone, selected karyotyping may be prudent.

### Symptomatic adults

Symptomatic adults can be tested for all disease categories mentioned previously as well as late onset genetic diseases following appropriate counseling and informed consent. These diseases include Parkinson’s disease, Alzheimer disease, and amyotrophic lateral sclerosis in worldwide populations and Huntington disease in Caucasians.

### Frequent diseases in specific populations

The 52 genetic diseases reported to be most frequent in specific populations listed in GeneTests have been incorporated to expedite optimal disease testing in regional laboratories (Additional file 1: Table S1F). Israeli laboratories will want to incorporate platforms with the most frequent Jewish mutations where 6,000,000 Israeli Jews reside, while laboratories in Sweden and Norway would include the Nordic mutations, labs in Quebec the French Canadian mutations, and Asian labs their regionally reported mutations. Worldwide, subpopulations of citizens within specific populations that typically select partners from their own ethnic groups would readily be served by incorporating the few frequent mutations for these diseases into the population wide screening test. These include Asian, Black, and Jewish United States citizens that each comprise at least 2% of the entire pan ethnic United States population (Additional file 1: Table S1F Group 3; Ref. [39]) and regional laboratories in central Pennsylvania, northeast Ohio, and Colorado where 200,000 Amish and Mennonite regional residents [0.08% of the total panethnic population] carry >50 specific gene mutations reported in these descendents of a few score of founders.

### Targeting frequent disease loci enriches clearly defined reportable results

Currently interpreting genomewide exome sequencing of all ~22,000 genes requires several times the cost of sequencing. Dr. Hruban reported “a human exome has on average almost 36,000 variants, 45 percent of which are not in an SNP database and about 100 of which can cause loss of function” [40]. He concluded, “The potential power of next-generation sequencing for clinical testing is substantial. It will be a while before it is brought fully to the clinic…” This agrees with Chun et al. who identified ~90 potential disease causing sequences identified by exome analysis of all ~22,000 genes in three individuals [5]. When testing 100,000 patients for the frequent autosomal recessive cystic fibrosis mutations with 99.9% test accuracy per gene, a positive carrier test would include 3445 correct answers and 103 incorrect answers (Table 3, Top; Ref. [20]). In contrast, for the rare fumarase deficiency gene locus with the same test accuracy a positive carrier test would not only include 26 correctly analyzed carriers but also 100 incorrect false positive carrier test results [Table 3, Bottom]. Maintaining the principle of testing the most frequent diseases listed from most to less frequent in each selected category provides an objective basis to select the lowest frequency of any tested disease in each category.

## Discussion

In summary. analyzing the 227 diseases affecting 263 loci that are each reported to affect at least 1 in 100,000 Caucasian individuals together include ~1.2% of all the 22,000 gene loci [~1 in 84], and ~9.1% [~1 in 11] of the >2500 listed clinically testable disease causing genes [1]. Based upon these frequencies, analyzing all >2500 known disease causing genes is estimated to identify ~11 potential disease causing genes. In contrast, testing the attached list of most frequent 227 disease loci in the United States [Additional file 1: Table S1, Additional file 2: Table S2 and Additional file 3: Table S3] is anticipated to decrease the number of candidate affected genotypes to be considered to ~0-2 per patient. This list of frequent diseases includes ~7 of 8 of McKusick’s (1982) estimated single locus Caucasian birth defects {[1.91/ (0.4% + 1.8%) = 87%] Table 1.3a, Ref. [41]} and ~6 of 7 Worldwide birth defects without region specific testing. [Derived from McKusick’s estimated 1.8% transmitted by Mendelian inheritance plus 0.4% chromosome abnormalities]. These most frequent diseases affect about 1 in 52 Caucasian newborns and 1 in 54 worldwide [Table 1.3.a,a’] These listed sites [Additional file 1: Table S1, Additional file 2: Table S2 and Additional file 3: Table S3] focus testing to the fewest most informative disease gene locations to achieve the highest possible test accuracy (Table 3; Refs. [20,42]). Because abnormal phenotypes are frequently seen in more than one genetic disease or chromosome abnormality, this targeted genomewide patient category testing approach can readily identify suspected and unsuspected abnormalities too numerous to test individually.

The 54 targeted chromosomal sites that would have identified *all* the microscopically visible aneuploid chromosome regions in our reported karyotypes reflect the efficacy of targeting the most frequent disease causing rearrangements. Currently microarrays and exome sequencing identify nearly all Genomewide normal and abnormal modifications. Genomewide platforms and computer programs can now be targeted to analyze only the most frequent disease causing sites [Additional file 1: Table S1, Additional file 2: Table S2 and Additional file 3: Table S3]. Platforms and computer programs that reveal physician selected patient categories and the diseases to be included will further enhance laboratory turnaround time [Figure 1A,B,C; Table 1]. Any selected platform category is likely to determine copy number. Upon further modification, rapid sequencing platforms and/or polymorphic or sequencing microarrays are anticipated to enable analyzing the most frequent reported disease causing mutations at fewer tested sites to most efficiently utilize all manufacturing, testing, interpretation, and counseling resources. Reporting only peer reviewed published mutations with their references would enable actionable results while minimizing inconclusive test risks.

The 13 frequent deletions reported in ~1 in 21 of the patients tested by microarrays were ~52-fold more frequent in this physician selected population than in the general population [1 in 1097; Additional file 3: Table S3B, Top)]. Although the relative contribution of each submitted patient category will be modified according to the physician’s specialty, substantial enrichment of abnormal genotypes is anticipated in all affected patient categories submitted for testing. This will improve upon total test accuracy.

Given the use and analysis of large genome data in clouds, companies are offering sufficient computing capability to analyze and store genomewide sequencing data for subsequent reanalysis. Given multiple reanalyses could be applied to the same data set as additional disease genes are discovered, disease gene mutations are cataloged, and new clinical information becomes available, stored genomewide data can be reanalyzed without resequencing. Laboratories that choose to provide this service can write additional programs to analyze all the data and distribute summaries of new findings to contributing physicians. All this requires prior patient understanding and agreement to receive updated information by continuing these analyses. The Perspective by Dr. Pyeritz addresses possible legal implications of reporting or of not reporting newly discovered genes [43]. Ongoing modifications to maintain optimal test platforms can be based upon additional identified disease genes, a change in individual observed disease frequency in sampled affected patients, ongoing test results, the geographical origin(s) of tested patients, and additional published causative mutations.

The U.S. Army Corps of Engineers Motto is, “*The difficult we do immediately. The impossible takes a little longer”*. Computer programs that reveal only physician selected gene results on the core panel can immediately optimize patient specific testing and minimize laboratory liability for unreported loci. Targeting and testing the most frequent genetic abnormalities on a single platform [Additional file 1: Table S1 A-G] will identify most clinically meaningful abnormal genotypes for any designated patient category. Testing all the confirmed disease associated genes with the most frequent disease core would provide ~10-fold less data than the total disease causing exome platform. The following applications can be considered immediately:

1. Testing the 54 most frequent chromosomal sites to identify most microscopically visible karyotypic abnormalities in fetal DNA in maternal circulation.
2. Targeting less than 64 frequent worldwide genomic abnormalities in the core list [Additional file 1: Table S1A,B] to readily identify the largest proportion of couples at-risk for affected fetuses worldwide.
3. Targeting ~257 listed genomic sites (Additional file 1: Table S1A-E) would identify most known genetic disease-causing mutations in abnormal children.
4. Developing targeted genomewide testing for the most frequent abnormalities including both single nucleotide mutations and gene aneuploidy on a single platform (Additional file 1: Table S1, Additional file 2: Table S2 and Additional file 3: Table S3) to optimize genomewide testing.
5. Computer programs written to only reveal each physician selected patient category and genes within it on any genomewide panel enables targeted testing with the fewest platforms.
6. Adding population-specific frequent disease mutations according to a testing laboratory’s geographical location.

## Conclusion

This principle of selecting and testing the most frequent genomewide disease causing abnormalities in ~1 of 8 known disease loci (1 of 84 total gene loci) is estimated to identify the genetic defect in ~7 of 8 reported abnormal newborn Caucasians. In contrast, this would eliminate ~8 to10 of ~10 Caucasian newborn gene sequences selected as abnormal that are actually normal variants identified when testing all ~4800 reported disease genes to search for the remaining 1 of 8 disease causing genes. Adopting this approach will minimize incorrect results while optimizing test accuracy, counseling, and reimbursement for a larger proportion of appropriate patients within available laboratory and reimbursement resources.

## Additional files

Additional file 1: Table S1. Most frequent genetic diseases by transmission category [25,44–47]. Additional file 2: Table S2A. 26 Abnormal karyotypic categories [48]. detected at 54 sites (Table 5) P27-29 [31]. Listed by frequency in POCs. **Table S2B.** Abnormal karyotypic categories detected at 54 sites. Listed by decreasing severity. Additional file 3: Table S3. Common submicroscopic aneuploid locI by chromosome location and category: [24,48]. Additional file 4: Table S4. Current platform detection capability [49–56].
