# Supplementary material for: Analyzing the most frequent disease loci in targeted patient categories optimizes disease gene identification and test accuracy worldwide
Source: J Transl Med. 2015 Jan 21;13:16. doi: 10.1186/s12967-014-0333-8 (PMC4312458; doi:10.1186/s12967-014-0333-8)
Supplement: Additional file 1: Table S1. — Most frequent genetic diseases by transmission category [25,44–47]. [file 12967_2014_333_MOESM1_ESM.doc]

**Additional file 1: Table S1.** **MOST FREQUENT GENETIC DISEASES BY TRANSMISSION CATEGORY [49]**

| **(A)** | |  | | **Autosomal Recessive [Affected = Homozygote]** | | ***# Genes*** | ***Affected*** | | | ***Heterozygote*** | | | |
| --- | --- | --- | --- | --- | --- | --- | --- | --- | --- | --- | --- | --- | --- |
|  | |  | | **Carrier = Heterozygote** | | ***Tested*** | ***Pop Freq*** | | | ***Pop Freq*** | | | |
|  | |  | |  | |  | **(q)2** | | | **(2pq)** | | | |
| 1 | | 1a(NB) | | Cystic fibrosis (caucasian) | |  | ~1/3,364 | | | ~1/29.5 | | | |
| 2 | | 2b,c | | Alpha-1-Antitrypsin Deficiency (caucasian) | |  | ~1/6,000 | | | ~1/38.2 | | | |
| 3 | | 3b | | DFNB1 - connexin 26 and connexin **30-50% AR-Testing GJB2** | |  | ~1/7,000 | | | ~1/42.3 | | | |
| 4 | | 4a(NB) | | Phenylketonuria (PKU) | |  | ~1/10,000 | | | ~1/50.5 | | | |
| 5 | | 5a(NB) | | 21-Hydroxylase-Deficient Congenital Adrenal Hyperplasia | |  | ~1/13,000 | | | ~1/57.5 | | | |
| 6 | | 6a | | Spinal muscular atrophy | |  | ~1/17,500 | | | ~1/66.7 | | | |
| 7 | | 7a-c | | Congenital Disorder of Glycosylation Type 1a - PMM2 | |  | ~1/20,000 | | | ~1/71.2 | | | |
| 8 | | 8a(NB) | | Medium chain acyl coenzyme A dehydrogenase | |  | ~1/28,500 | | | ~1/84.9 | | | |
| 9 | | 9c | | Leber Hereditary Optic Neuropathy - **70%** | |  | ~1/29,250 | | | *~1/85.9* | | | |
| 10 | | 10c | | Spastic Paraplegia 7 - SPG7 | |  | ~1/32,500 | | | *~1/90.6* | | | |
| 11 | | 11a,b | | Very Long Chain Acyl-Coenzyme A Dehydrogenase Deficiency | |  | ~1/30,000 | | | ~1/87.1 | | | |
| 12 | | 12a | | Smith-Lemli-Opitz Syndrome | |  | ~1/30,000 | | | ~1/87.1 | | | |
| 13 | | 13c | | Wilson Disease | |  | ~1/30,000 | | | ~1/87.1 | | | |
| 14 | | 14a,b | | Friedreich Ataxia (affects minors) -trinucleotide repeat | |  | ~1/37,500 | | | *~1/97.4* | | | |
| 15 | | 15c | | Oculocutaneous Albinism Type 2 | |  | ~1/39,000 | | | *~1/99.2* | | | |
| 16 | | 16b | | Oculocutaneous Albinism Type 1 - | | 2 Genes | ~1/40,000 | | | *~1/100.5* | | | |
| 17 | | 17b | | Leber Congenital Amaurosis - **4 of 15 Genes for ~49%+** | | 4 Genes | ~1/41,500 | | | *~1/204.7+* | | | |
| 18 | | 18b | | Hemophagocytic Lymphohistiocytosis - **3 of 5 Genes for 71%** | | 5 Genes | ~1/50,000 | | | *~1/158.2+* | | | |
| 19 | | 19a | | Zellweger Syndrome - **2 Genes detect 74%+** | | 2 Genes | ~1/50,000 | | | *~1/151.8+* | | | |
| 20 | | 20c | | Pendred Syndrome/DFNB4 - SLC26A4 | |  | ~1/50,000 | | | ~1/112.3 | | | |
| 21 | | 21b | | Cerebrotendinous Xanthomatosis - 1 mutation | |  | ~1/50,000 | | | ~1/112.3 | | | |
| 22 | | 22c | | Myotonia Congenita **AR** | |  | ~1/50,000 | | | ~1/112.3 | | | |
| 23 | | 23a-c | | Short-Chain Acyl-CoA Dehydrogenase Deficiency | |  | ~1/50,000 | | | ~1/112.3 | | | |
| 24 | | 24c | | Glycogen Storage Disease Type VI | | 2 Genes | ~1/50,000 | | | ~1/112.3 | | | |
| 25 | | 25a(NB) | | Citrullinemia Type I | |  | ~1/57.000 | | | *~1/119.9* | | | |
| 26 | | 26a | | Pompe Disease -GSD II | |  | *~1/60,000 | | | *~1/123.0* | | | |
| 27 | | 27a(NB) | | Biotinidase Deficiency | |  | ~1/61,000 | | | *~1/124.0* | | | |
| 28 | | 28c | | *LIS1*-Associated Lissencephaly - 20% inherited | |  | ~1/62,500 | | | *~1/125.5* | | | |
| 29 | | 29a(NB) | | Argininosuccinate Lyase Deficiency | |  | ~1/70,000 | | | *~1/132.8* | | | |
| 30 | | 30a(NB) | | Galactosemia | |  | **~1/70,000 | | | *~1/132.8* | | | |
| 31 | | 31a(NB) | | Methylmalonic Acidemia - **3 of 5 Genes Tested** | | 3 Genes | ~1/80,000 | | | *~1/141.9* | | | |
| 32 | | 32c | | Ehlers-Danlos Syndrome, Kyphoscoliotic Form | |  | ~1/100,000 | | | *~1/158.6* | | | |
| 33 | | 33b | | Limb-Girdle Muscular Dystrophy Type 2A - Calpainopathy | |  | ~1/100,000 | | | *~1/158.6* | | | |
| 34 | | 34b | | Hypophosphatasia - autosomal recessive | |  | ~1/100,000 | | | *~1/158.6* | | | |
| 35 | | 35b | | Diastrophic Dysplasia (Dwarfism) | |  | ~1/100,000 | | | *~1/158.6* | | | |
| 36 | | 36b | | Arylsulfatase A Deficiency | |  | ~1/100,000 | | | *~1/158.6* | | | |
| 37 | | 37a | | Polycystic Kidney Disease, Autosomal Recessive | | 3 Genes | ~1/125,000 | | | *~1/177.3* | | | |
|  | |  | |  | | **TOTAL** | **TOTAL=1/668** | | | **1/2.48 = ~2/5** | | | |
|  | |  | | Couples = (carrier frequency)2 added together | |  | **Ave = 1/24,700** | | | ***Couples 1/174*** | | | |
|  | |  | | **Late Onset** | |  |  | | |  | | | |
| 38 | | 38 | | HFE-Assoc. Hemochromatosis -3 mutations (Caucasian) | |  | 1/200-1/400 | | | 1/8.66 | | | |
| 39 | | 39 | | Parkinson Disease - Testing for 1 gene (PARK 2)-50% pickup | |  | 1/200 >55yo | | | 1/7… | | | |
|  | |  | |  | |  |  | | |  | | | |
|  | |  | | **Multiple Genes** (excluded from Table calculations) | |  |  | | |  | | | |
|  | | 40 | | Congenital Myasthenic Syndromes - [testing 5 of 11 genes~45%] | | 11 Genes | 1/24,000 | | | 1/77.45 | | | |
|  | | 41 | | Usher Syndrome type 1 - [5 of 8 gene loci tested] | | 8 Genes | 1/22,700 | | | 1/75.33 | | | |
|  | |  | | - 50% deaf-blindness, 3%-6% deafness | |  |  | | |  | | | |
|  | | 42 | | Peroxisome Biogenesis, Zellweger - **12 Genes ~95%** | | 12 genes | 1/50,000 | | | *1/111.8* | | | |
|  | |  | |  | |  |  | | |  | | | |
|  | |  | |  | |  |  | | |  | | | |
| **(B)** | |  | | **Frequent X-linked-Lower Maternal Carrier Frequency** | |  |  | | | **Heterozygotes** | | | |
|  | |  | |  | |  |  | | |  | | | |
|  | | **Recessive Affected = All Hemizygotes + Homozygotes.** | | | |  | **[(q +q2)] (C,D,E)** | | | **(2pq)** | | | |
|  | | **Dominant Affected = All Hemizygotes + Heterozygotes + Homozygotes.** | | | | | **[(q + 2pq +q2] (D,E)** | | | **(2pq)** | | | |
| 40 | | 1 | | FRMD7-Related Infantile Nystagmus | |  | 1/5,000 | | | ~1/2,500 | | | |
| 41 | | 2 | | Hemophilia A | |  | 1/10,000 | | | ~1/5,000 | | | |
| 42 | | 3 | | X-Linked Adrenal Hypoplasia Congenita | |  | 1/12,500 | | | ~1/6,250 | | | |
| 43 | | 4 | | X-linked Juvenile Retinoschisis | |  | 1/15,000 | | | ~1/7,500 | | | |
| 44 | | 5 | | Duchenne muscular dystrophy | |  | 1/11,236 (G) | | | ~1/5,618 | | | |
| 45 | | 6 | | Becker muscular dystrophy | |  | 1/36,900 | | | ~1/18,450 | | | |
| 46 | | 7 | | Hemophilia B | |  | 1/20,000 | | | ~1/10,000 | | | |
| 47 | | 8 | | X-Linked Adrenoleukodystrophy | |  | 1/30,000 | | | ~1/15,000 | | | |
| 48 | | 9 | | Lowe Syndrome | |  | 1/30,000 | | | ~1/15,000 | | | |
| 49 | | 10 | | Androgen Insensitivity Syndrome | |  | 1/35,000 | | | ~1/17,500 | | | |
| 50 | | 11 | | Spastic Paraplegia Type 1 - L1 Syndrome - LICAM gene | |  | 1/30,000 | | | ~1/15,000 | | | |
| 51 | | 12 | | Coffin-Lowry Syndrome -- X-linked dominant | |  | 1/45,000 | | | *~1/67,500* | | | |
| 52 | | 13 | | Choroideremia - CHM Gene | |  | 1/50,000 | | | ~1/25,000 | | | |
| 53 | | 14 | | Fabry Disease | |  | 1/50,000 | | | ~1/25,000 | | | |
| 54 | | 15 | | X-linked myotubular Myopathy MTM1 Gene | |  | 1/50,000 | | | ~1/25,000 | | | |
| 55 | | 16 | | Ocular Albinism, X-Linked | |  | 1/50,000 | | | ~1/25,000 | | | |
| 56 | | 17 | | Alport - Collagen 4A5 - 1 gene tested | |  | 1/62,500 | | | ~1/31,250 | | | |
| 57 | | 18 | | Retinitis Pigmentosa | | 2 Genes | 1/29,487 | | | ~1/14,744 | | | |
| 58 | | 19 | | X-Linked Severe Combined Immunodeficiency | |  | 1/75,000 | | | ~1/37,500 | | | |
| 59 | | 20 | | Double Cortex Syndrome - X-linked dominant | |  | 1/85,000 | | | *~1/127,500* | | | |
| 60 | | 21 | | Fabry Disease | |  | 1/100,000 | | | ~1/50,000 | | | |
| 61 | | 22 | | Menkes/ATP7A-Related Copper Transport Dis | |  | 1/100,000 | | | ~1/50,000 | | | |
|  | |  | |  | | **TOTAL** | **1/1065** | | | ***1/546*** | | | |
|  | |  | |  | | **AVE** | **1/23,440** | | | ***1/12,012*** | | | |
|  | |  | |  | |  |  | | |  | | | |
| 62 | | 23 | | Fragile X - trinucleotide repeat analysis | |  | 1/5,000 | | | 1/1250 | | | |
| 63 | | 24 | | MECP2-Rett Syndrome - test in fetus >99% de novo | |  | 1/8,500 | | | *<1/850,000* | | | |
|  | |  | |  | |  |  | | |  | | | |
| **(C)** | |  | | **Frequent Autosomal Dominant:** | |  |  | | | **2pq**~(2pq + q2) | | | |
|  | |  | | **Affected = Heterozygote + Homozygote** | |  | **(2pq + q2 )** | | | ~2q because | | | |
|  | |  | |  | |  |  | | | q2 < 1/100,000) | | | |
| 64 | | 1 | | Polycystic Kidney Disease, Autosomal Dominant | |  | 1/700 | | | 1/700 | | | |
| 65 | | 2 | | BRCA1 - adult onset | |  | 1/750 | | | 1/750 | | | |
| 66 | | 3 | | Duane Syndrome | |  | 1/1,000 | | | 1/1000 | | | |
| 67 | | 4 | | Thoracic Aortic Aneurysms and Dissections -5 Genes | | 2 Genes | 1/1166 X .16 | | | 1/7,288 | | | |
| 68 | | 5 | | Noonan Syndrome - 3 gene test for ~75% | | 4 Genes | 1/1750 X .75 | | | 1/2333 = | | | |
| 69 | | 6 | | Early-Onset Familial Alzheimer Disease - adult onset 40-59 yo | |  | 1/2427= | | | 1/2427= | | | |
| 70 | | 7 | | Neurofibromatosis 1 - adult onset | |  | 1/3,000 = | | | 1/3,000 = | | | |
| 71 | | 8 | | *SHOX*-Related Haploinsufficiency - pseudoautosomal | |  | 1/4,000 | | | 1/4,000 = | | | |
| 72 | | 9 | | Long QT Syndrome, Autosomal Dominant | |  | 1/5,000 | | | 1/5000 = | | | |
| 73 | | 10 | | DFNA2 Nonsyndromic Hearing Loss -KCNQ4 Gene - Test ~5% | |  | 1/5,720 | | | *1/5,720* | | | |
| 74 | | 11 | | Tuberous Sclerosis Complex - 2/3 de novo | | 2 Genes | 1/5,800 | | | 1/5,800 | | | |
| 75 | | 12 | | Charcot-Marie-Tooth Neuropathy Type 1A | |  | 1/5,714 | | | 1/5,833 | | | |
| 76 | | 13 | | Hereditary Neuropathy with Liability to Pressure Palsies | |  | 1/35,000 | | | 1/35,000 | | | |
| 77 | | 14 | | Marfan Syndrome - 25% de novo | |  | 1/7,500 | | | 1/7,500 | | | |
| 78 | | 15 | | Spastic Paraplegia Type 4 | |  | 1/8,000 | | | 1/8,000 | | | |
| 79 | | 16 | | Myotonic Dystrophy Type 2 | |  | 1/8,000 | | | 1/8,000 | | | |
| 80 | | 17 | | Amyotrophic Lateral Sclerosis - Lou Gehrig's Disease | |  | 1/18,750 | | | *1/18750* | | | |
| 81 | | 18 | | Stickler Syndrome - Test 2 Genes | | 4 Genes | 1/8,250 | | | 1/8,250 | | | |
| 82 | | 19 | | Hereditary Hemorrhagic Telangiectasia - 2 Genes for ~90% | | 3 Genes | 1/10,000 | | | 1/10,000 | | | |
| 83 | | 20 | | Charge Syndrome - 1 Gene | |  | 1/10,000 | | | 1/10,000 | | | |
| 84 | | 21 | | Charcot-Marie-Tooth Neuropathy Type 2A2 -1 gene | |  | 1/10000 | | | *1/10,000* | | | |
| 85 | | 22 | | Wilms Tumor - WAGR - most de novo | |  | 1/12,000 | | | 1/12,000 | | | |
| 86 | | 23 | | Ehlers-Danlos Syndrome, Hypermobility Type | |  | 1/12,500 | | | 1/12,500 | | | |
| 87 | | 24 | | Dilated Cardiomyopathy - 4 Genes for ~ 20% | | 4 genes | 1/13,500 | | | 1/13,500 | | | |
| 88 | | 25 | | Osteogenesis Imperfecta | |  | 1/15,000 | | | 1/15,000 | | | |
| 89 | | 26 | | Retinoblastoma | |  | 1/17,500 | | | 1/17,500 | | | |
| 90 | | 27 | | Myotonic Dystrophy Type 1 - trinucleotide repeat | |  | 1/20,000 | | | 1/20,000 | | | |
| 91 | | 28 | | Ehlers-Danlos Syndrome, Classic Type - test 2 gene-50% | | 2 Genes | 1/20,000 X .50 | | | 1/40,000 | | | |
| 92 | | 29 | | Li-Fraumeni Syndrome ~ 95% | |  | 1/20,000 X .95 | | | 1/21,053 | | | |
| 93 | | 30 | | APC-Associated Polyposis Conditions >80% inherited | |  | 1/25,000 | | | 1/25,000 | | | |
| 94 | | 31 | | Waardenburg Syndrome, Type 1 - nearly all inherited | |  | 1/30,000 | | | 1/30,000 | | | |
| 95 | | 32 | | Multiple Endocrine Neoplasia Type 1 - 10% de novo | |  | 1/30,000 | | | 1/30,000 | | | |
| 96 | | 33 | | Muenke Syndrome | |  | 1/30,000 | | | 1/30,000 | | | |
| 97 | | 34 | | Juvenile Polyposis Syndrome 25% de novo; 2 Genes ~52% | | 2 Genes | 1/30,000 X .52 | | | 1/57692 | | | |
| 98 | | 35 | | Treacher Collins Syndrome - 60% de novo | |  | 1/30,000 | | | 1/30,000 | | | |
| 99 | | 36 | | Optic Atrophy, Type 1 - OPA1 | |  | 1/30,000 | | | 1/30,000 | | | |
| 100 | | 37 | | Hypochondroplasia - >99.9% de novo | |  | 1/27,500 | | | 1/27,500 | | | |
| 101 | | 38 | | Achondroplasia FGFR3 - 80% de novo | |  | 1/27,500 | | | 1/27,500 | | | |
| 102 | | 39 | | Multiple Endocrine Neoplasia Type 2 | |  | 1/35,000 X .98 | | | 1/35,714 | | | |
| 103 | | 40 | | Spinocerebellar Ataxia Type 1 - ATXN1 - adult onset -trinucleotide | | | 1/35,000 | | | 1/35,000 | | | |
| 104 | | 41 | | Thanatophoric Dysplasia - FGFR3 | |  | 1/35,000 | | | 1/35,000 | | | |
| 105 | | 42 | | Saethre-Chotzen Syndrome - TWIST 1 Gene | |  | 1/37,500 | | | 1/37,500 | | | |
| 106 | | 43 | | Epidermolysis Bullosa Simplex - 2 Genes ~88% | | 2 Genes | 1/40,000 X .88 | | | *1/45,454* | | | |
| 107 | | 44 | | Malignant Hyperthermia Susceptibility 6 Genes, 1 Tested ~75% | |  | 1/40,000 X .75 | | | *1/53,333* | | | |
| 108 | | 45 | | Branchiootorenal Spectrum Disorders - 3 Genes, Test 2 ~42.5% | |  | 1/40,000 X .425 | | | *1/94118* | | | |
| 109 | | 46 | | Spastic Paraplegia 3A - - >95% inherited | |  | 1/8,000-1/80,000 | | | 1/44,000 | | | |
| 110 | | 47 | | Optic Atrophy Type 1 | |  | 1/50,000 | | | *1/50,000* | | | |
| 111 | | 48 | | Nail-Patella Syndrome - LMX18 Gene ~95% | |  | 1/50,000 X .95 | | | *1/52,632* | | | |
| 112 | | 49 | | Charcot-Marie-Tooth Neuropathy Type 1B childhood onset | |  | 1/50,000 | | | 1/50,000 | | | |
| 113 | | 50 | | Charcot-Marie-Tooth Neuropathy MFN2 Gene - late onset | |  | 1/50,000 | | | *1/50,000 =* | | | |
| 114 | | 51 | | Spastic Paraplegia 8 | |  | *1/52750 =* | | | *1/52750 =* | | | |
| 115 | | 52 | | Fascioscapulohumeral Muscular Dys -D4Z4 deletion-80% de novo | | | 1/55,000 | | | 1/55,000 | | | |
| 116 | | 53 | | Cornelia de Lange Syndrome - most de novo | | 3 Genes | 1/55,000 X .66 | | | *1/55,000* | | | |
| 117 | | 54 | | Campomelic Dysplasia -SOX9 Gene -most de novo | |  | 1/60,000 | | | *1/60,000* | | | |
| 118 | | 55 | | Aniridia - 30% de novo | |  | 1/70,000 X 70% | | | *1/100,000* | | | |
| 119 | | 56 | | Hydroxymethylbilane Synthase (HMBS) Deficiency | |  | *1/75,000 =* | | | *1/75,000 =* | | | |
| 120 | | 57 | | Alagille Syndrome - 90% in 2 genes | | 2 Genes | 1/77,777 | | | 1/77,777 | | | |
| 121 | | 58 | | Charcot-Marie-Tooth Disease Type 2B - DNM2 Gene | |  | 1/98,000 | | | 1/98,000 = | | | |
| 122 | | 59 | | Holt-Oram Syndrome - 15% inherited | |  | 1/100,000 | | | 1/100,000 | | | |
| 123 | | 60 | | Familial Transthyretin Amyloidosis | |  | 1/100,000 U.S. | | | *1/100,000 =* | | | |
|  | |  | |  | | **TOTAL** | **1/123** | | | ***1/123*** | | | |
|  | |  | |  | | **Average** | **1/7395…** | | | ***1/7393…*** | | | |
|  | |  | | **Multiple Genes** (Excluded from calculations) | |  |  | | |  | | | |
|  | | 61 | | Familial Hypertrophic Cardiomyopathy - 6 of 12 Genes ~93% | | 6 Genes | 1/500 X.93 | | | 1/538 | | | |
|  | | 62 | | Arrhythmogenic Right Ventricular Dysplasia/Cardiomyopathy, | | 8 Genes | 1/1000-1/1250 | | | 1/1125 = | | | |
|  | | 63 | | Retinitis Pigmentosa | | 6 Genes | 1/138,000 | | | 1/828,000 = | | | |
|  | | 64 | | Diamond-Blackfan Anemia | | 9 Genes | 1/100,000-1/200,000 | | | 1/1,350,000 = | | | |
|  | |  | | **Mild** (excluded from Table 4 calculations) | |  |  | | |  | | | |
|  | | 65 | | Enlarged Parietal Foramina 1,2 -mild to severe; ~90% penetrant | | 2 Genes | ~1/32,500 | | | ~1/32,500 | | | |
|  | |  | |  | |  |  | | |  | | | |
| **(D)** |  | | **Y-linked (Paternal) Inheritance** | |  | | | |  | | |  | |
|  |  | | **Affected - Hemizygous male** | |  | | | |  | | |  | |
| 124 | 1 | | 46,XX Testicular Disorder of Sex Development-SRY gene ~80% | | 1 Gene | | | | 1/25000 | | |  | |
| 125 | 2 | | AZFa,b,c Azoospermia - microdeletions ~10% of total | | 3 Genes | | | | 1/25,000 | | |  | |
|  |  | |  | | **TOTAL** | | | | **1/12,500** | | |  | |
| **(E)** | |  | | **Mitochondrial (Maternal) Inheritance** | |  | |  | | |  | |  |
|  | | 1 | | **Mitochondrial DNA-Associated Leigh Syndrome and NARP** | |  | | 1/10000-1/86000 | | |  | |  |
|  | | 2 | | **Mitochondrial Deletion Syndromes** | | >12genes | | 1 in 8500 | | |  | |  |
|  | |  | |  | |  | |  | | |  | |  |
| **(F)** | |  | | **Frequent in a Specific Population** | |  | |  | | |  | |  |
|  | |  | |  | |  | |  | | |  | |  |
|  | |  | | **Caucasian- ~77% Americans; 1.7 billion worldwide** | |  | |  | | |  | |  |
| 126 | | 1 | | HFE-Hereditary Hemochromatosis -3 mutations - late onset | |  | | 1/300 | | |  | |  |
| 127 | | 2 | | Cystic fibrosis | |  | | 1 in 3363 | | |  | |  |
| 128 | | 3 | | Alpha-1-Antitrypsin Deficiency | |  | | 1 in 6000 | | |  | |  |
| 129 | | 4 | | Huntington Disease -late onset | |  | | ~1/20,000 | | |  | |  |
|  | |  | |  | |  | |  | | |  | |  |
|  | |  | | **African regions - ~12.6% U.S. Americans** | |  | |  | | |  | |  |
| 130 | | 5 | | Sickle Cell Disease | |  | | 1/12 parts of Africa | | |  | |  |
| 131 | | 6 | | Glycogen Storage Disease Type II | |  | | 1/14,000 Black U.S. | | |  | |  |
| 132 | | 7 | | Oculocutaneous Albinism Type 2 | |  | | 1/4,750 Africans | | |  | |  |
|  | |  | |  | |  | |  | | |  | |  |
|  | |  | | **Southeast Asia/Mediterranean** | |  | |  | | |  | |  |
| 133 | | 8 | | Alpha-Thalassemia - Southeast Asia | |  | | 1/333 Hb Barts + Hb H | | |  | |  |
| 134 | | 9 | | Beta-Thalassemia - Southeast Asia and Meditedrranean | |  | | 1/200 in Cyprus | | |  | |  |
| 135 | | 10 | | Brugada (Long QT) Syndrome -common worldwide | |  | | 1/2,000 S.E. Asians | | |  | |  |
|  | |  | |  | |  | |  | | |  | |  |
|  | |  | | **Asian -4.8% Asian American** | |  | |  | | |  | |  |
| 136 | | 11 | | Ataxia with Vitamin E Deficiency | |  | | 1/15,000 Japanese Is. | | |  | |  |
| 137 | | 12 | | Citrin Deficiency | |  | | 1/17,000 Japan | | |  | |  |
| 138 | | 13 | | Werner Syndrome | |  | | 1/30,000 Japan | | |  | |  |
| 139 | | 14 | | Primary Ciliary Dyskinesia – 2 Genes | |  | | 1/16,000 Japan, U.S. | | |  | |  |
| 140 | | 15 | | Oculopharyngeal Muscular Dystrophy | |  | | 1/10,000 Japan | | |  | |  |
|  | |  | |  | |  | |  | | |  | |  |
|  | |  | | **Jewish-2% Jewish American** | |  | |  | | |  | |  |
| 141 | | 16 | | Arylsulfatase A Deficiency | |  | | 1/100 Ashkenazi | | |  | |  |
| 142 | | 17 | | Familial Mediterranean Fever - throughout Mideast | |  | | 1/100 Ashkenazi | | |  | |  |
| 143 | | 18 | | Gaucher Disease | |  | | 1/855 Ashkenazi | | |  | |  |
| 144 | | 19 | | Inclusion Body Myopathy 2 | |  | | 1/900 Iranian Jews | | |  | |  |
| 145 | | 20 | | Dysferlinopathy | |  | | 1/1300 Libyan Jews | | |  | |  |
| 146 | | 21 | | Early-Onset Primary Dystonia (DYT1) | |  | | 1/6,000 Ashkenazi | | |  | |  |
| 147 | | 22 | | Tay Sachs Disease | |  | | 1/3,600 Ashkenazi | | |  | |  |
| 148 | | 23 | | Familial Dysautonomia (HSAN III) | |  | | 1/3,700 Ashkenazi | | |  | |  |
| 149 | | 24 | | Glycogen Storage Disease Type III | |  | | 1/5,400 North African | | |  | |  |
| 150 | | 25 | | Bloom Syndrome | |  | | 1/4,000 Ashkenazi | | |  | |  |
| 151 | | 26 | | Oculopharyngeal Muscular Dystrophy | |  | | 1/600 Israeli Bukhara | | |  | |  |
| 152 | | 27 | | Canavan Disease | |  | | ~1/10,000 Ashkenazi | | |  | |  |
| 153 | | 28 | | Fanconi anemia - FANCA gene | |  | | 1/40,000 Ashkenazi | | |  | |  |
| 154 | | 29 | | Niemann-Pick (Source: Lazarin et al, 2012) | |  | | 1/40,000 Ashkenazi | | |  | |  |
| 155 | | 30 | | Bloom Syndrome (Source: Lazarin et al, 2012) | |  | | 1/40,000 Ashkenazi | | |  | |  |
| 156 | | 31 | | Mucolipidosis IV | |  | | 1/50,000 Ashkenazi | | |  | |  |
|  | |  | |  | |  | |  | | |  | |  |
|  | |  | | **French-Canadian-0.66% U.S.** | |  | |  | | |  | |  |
|  | | 32 | | Oculopharyngeal Muscular Dystrophy | |  | | 1/1000 French Canada | | |  | |  |
|  | | 33 | | Ataxia with Oculomotor Apraxia Type 2 | |  | | 1/3,400 French Canada | | |  | |  |
|  | | 34 | | Mucolipidosis II | |  | | 1/6184 Quebec | | |  | |  |
|  | |  | |  | |  | |  | | |  | |  |
|  | |  | | **Nordic** | |  | |  | | |  | |  |
|  | | 35 | | Hydroxymethylbilane Synthase (HMBS) Deficiency | |  | | 1/10,000 Sweden | | |  | |  |
|  | | 36 | | Udd Distal Myopathy | |  | | 9/100,000 Finland | | |  | |  |
|  | | 37 | | Glycine Encephalopathy | |  | | 1/12,000 Finland | | |  | |  |
|  | | 38 | | *SCN9A*-Related Inherited Erythromelalgia | |  | | 1/50,000 Norway | | |  | |  |
|  | | 39 | | MELAS (Mitochondrial Encephalomyopathy) | |  | | 1/6,000 Finland | | |  | |  |
|  | | 40 | | Familial Hyperinsulinism (FHI) | |  | | 1/2,500 Finland | | |  | |  |
|  | |  | |  | |  | |  | | |  | |  |
|  | |  | | **Middle Eastern** | |  | |  | | |  | |  |
|  | | 41 | | Phenylketonuria (listed above) | |  | | 1/2,600 Turks | | |  | |  |
|  | | 42 | | Hepatic Veno-Occlusive Disease with Immunodeficiency | |  | | 1/2,500 Aus.Lebanese | | |  | |  |
|  | | 43 | | Glycogen Storage Disease Type III | |  | | 1/3,100 Faroese | | |  | |  |
|  | | 44 | | Familial Hyperinsulinism (FHI) | |  | | 1/2,500 Saudi Arabia | | |  | |  |
|  | |  | |  | |  | |  | | |  | |  |
|  | |  | | **Amish/Mennonite -0.08% U.S. selected from >50 genes** | |  | |  | | |  | |  |
|  | | 45 | | Maple Syrup Urine Disease | |  | | 1/380 in Mennonites | | |  | |  |
|  | | 46 | | Nemaline Myopathy | |  | | 1/500 Amish | | |  | |  |
|  | | 47 | | Cohen Syndrome | |  | | 1/500 Amish | | |  | |  |
|  | | 48 | | McKusick-Kaufman Syndrome | |  | | 1/10,000 Amish | | |  | |  |
|  | |  | |  | |  | |  | | |  | |  |
|  | |  | | **Other Individual Populations** | |  | |  | | |  | |  |
|  | | 49 | | Low γ-GT Familial Intrahepatic Cholestasis | |  | | 1:160 Ittoqqortoormiit | | |  | |  |
|  | | 50 | | Trimethylaminuria -1 gene, 30 mutations | |  | | 1/325 in New Guinea | | |  | |  |
|  | | 51 | | 21-Hydroxylase-Deficient Congenital Adrenal Hyperplasia | |  | | 1:300 Alaskan Eskimos | | |  | |  |
|  | | 52 | | Congenital Cataracts, Facial Dysmorphism, and Neuropathy | |  | | 1/800 Rudari Gypsies | | |  | |  |
|  | | 53 | | Fryns Syndrome | |  | | 1/14,000 in France | | |  | |  |
|  | | 54 | | Cystinosis | |  | | 1/26,000 in Brittany | | |  | |  |
|  | | 55 | | X-Linked Dystonia-Parkinsonism | |  | | 1/6,000 Capiz Is. | | |  | |  |
|  | |  | |  | |  | |  | | |  | |  |
| **(G)** | |  | | **Complex Inheritance or Testing** | |  | |  | | |  | |  |
|  | | 1 | | Anophthalmia / Microphthalmia | |  | | 1/6700-1/25,000 | | |  | |  |
|  | | 2 | | von Willebrand Disease ~65% by sequencing | |  | | 1/10,000 severe | | |  | |  |
|  | | 3 | | Prothrombin Thrombophilia | |  | | 1/10,000 | | |  | |  |
|  | | 4 | | Craniofacial Microsomia - frequently de novo | |  | | 1/5600-1/26,550 | | |  | |  |
|  | | 5 | | Hirschsprung Disease - 50% de novo; 6 genes for ~93% | | 6 Genes | | 1/5,000 | | |  | |  |
|  | | 6 | | Gonadotropin-Releasing Hormone Def ~70% doe novo | | 11 Genes | | 1/10000-1/86000 | | |  | |  |
|  | | 7 | | Kallmann Syndrome - 6 X-linked Genes ~ 30%; + AD + AR | | 6 Genes | | 1/16,000-1/100,000 | | |  | |  |
|  | | 8 | | Retinitis Pigmentosa - AD, AR, X-linked | | 22 Genes | | 1/3,500-1/4,000 | | |  | |  |
|  | | 9 | | Bardet-Biedl Syndrome - 5 Genes = 57% | | 14 Genes | | 1/100,000 | | |  | |  |
|  | | 10 | | Holoprosencephaly - karyotyping + test 5 genes | | 8 Genes | | 1/250 embryos | | |  | |  |
|  | | 11 | | Emery-Dreifuss Muscular Dys.- X linked | | 2 Genes | | 1/100,000 | | |  | |  |
|  | | 12 | | FGFR-Related Craniosynostosis Syndromes | | 8 Disorders | | 1/2,000-1/2,500 | | |  | |  |
|  | |  | | AD - *variable penetrance and expressivity* | |  | |  | | |  | |  |
|  | |  | | **Poor detection rate**: | |  | |  | | |  | |  |
|  | | 13 | | MAPT-Related Disorders - detect 1% - 10%, AD, late onset | |  | | 1/17,000 | | |  | |  |
|  | | 14 | | Common Variable Immune Deficiency - 50% de novo | |  | | 1/30,000 | | |  | |  |
|  | |  | | *(A) De Grouchy and Turleau,1984. [45]* | |  | |  | | |  | |  |
|  | |  | | *(B) Vogel and Motulsky, 1986; [23]* | |  | |  | | |  | |  |
|  | |  | | *(C) Thompson MW, et.al., 1991, page 78. [46]* | |  | |  | | |  | |  |
|  | |  | | *(D) Nussbaum RL, et.al., 2007, pp. 195.[47]* | |  | |  | | |  | |  |
|  | |  | | *(E) Jorde et.al,. Medical Genetics. 3rd Edition, 2003, Pp.98.[48]* | |  | |  | | |  | |  |

**Table S1. Legend.**

**CORE LIST OF MOST FREQUENT GENETIC DISEASE GENES.**

**with Affected Frequencies Exceeding 1 in 100,000 People Worldwide**

**in Worldwide and in Specific Populations**

*Frequencies for each disease category are listed according to the frequency in the general population.* DNA laboratories report genotypes based upon homozygous, heterozygous, and hemizygous results. To expedite analysis, category specific heterozygous frequencies were calculated according to the mathematically derived values at the top of each section.

Molecular Diagnostic laboratories report genotypes based upon homozygous, heterozygous, and hemizygous results. To expedite analysis, category specific heterozygous frequencies (Table 1,A,B,C Column G) were calculated according to the mathematical equations at the top of each section. Given the frequencies to two allele categories p and q that result in normal and mutant phenotypes at each unique autosomal disease locus, then p + q = 1 includes all possible normal and abnormal alleles. For autosomal disease, the frequencies of these alleles at Hardy-Weinberg equilibrium are p2 +2pq + q2. For autosomal recessive disease, the frequency of affected patients is q2 and the frequency of heterozygous carriers is 2pq. When q is small with respect to p, the frequency of all patients with abnormal alleles is ~2q. For autosomal dominant diseases with an abnormal allele frequency of q, heterozygotes and homozygotes are both affected. Typically homozygotes are more severely affected than heterozygotes. The frequency of heterozygotes (2pq) is approximately equal to the total frequency of affected patients (2pq + q2) when q is small.

For X-linked alleles, males are hemizygous with 1 allele on their single X chromosome and females have two alleles carried by each of their X chromosomes. For X-linked dominant disease, all patients with at least one disease allele q are affected: hemizygous males with 1 q allele, heterozygous females with 1q allele, and homozygous females with 2q alleles. When affected patients have a reproductive fitness of 1 (F=1), ~2 times as many females are affected as males. For X-linked recessive alleles, hemizygous males with a single q allele are affected and homozygous females with 2q alleles are affected. Heterozygous females are usually unaffected or only mildly affected. When mildly affected, the severity is dependent upon the proportion of normal X chromosomes randomly inactivated in the affected tissue. Females affected with an X-linked recessive disease often have 45,X Turner syndrome.
