# Supplementary material for: Analyzing the most frequent disease loci in targeted patient categories optimizes disease gene identification and test accuracy worldwide
Source: J Transl Med. 2015 Jan 21;13:16. doi: 10.1186/s12967-014-0333-8 (PMC4312458; doi:10.1186/s12967-014-0333-8)
Supplement: Additional file 2: Table S2A. — 26 Abnormal karyotypic categories [48]. detected at 54 sites (Table 5) P27-29 [31]. Listed by frequency in POCs. Table S2B. Abnormal karyotypic categories detected at 54 sites. Listed by decreasing severity. [file 12967_2014_333_MOESM2_ESM.doc]

**Additional file 2: TABLE S2A. 26 Abnormal Karyotypic Categories [50]**

**Detected at 54 Sites (Table 8)**

**Listed by Frequency in POCs.**

***(POC + Newborn = population frequency; CVS/Amnio=consented to procedure)***

***All bold results occur in frequencies of at least 1 in 5,000 positive prenatal samples***

***And 1 in 20,000 positive newborns.***

***54 Sites* detect all aneuploid loci.** (Lebo et al., 2013)

**Sample Source: Products(1) CVS(2/1) Amniocentesis(2/1) Newborns(3)**

**of Conception 1987-95 SF/ 1970-95 SF/ 1986**

**2002-2013 OH 2007-13 OH 2002-2013 OH Seattle**

**------------------------------------------------------------------------------**

**% ABNORMAL 50% 3.1%// 2.3%// 0.6%**

**33.0% 7.2%**

**------------------------------------------------------------------------------**

**TOTAL TESTED: 1449 (1) 5,134 (2)// 21,288 (2)// 54,749(3)**

**82 (1) 2,598 (1)**

**-------------------------------------------------------------------------------**

**45,X – Turner 7.9% 0.29%// 4.90% 0.18%// 0.40% 0.040%**

***Trisomy 16 6.2%*** *0.00%// 0.00%* *0.00%****// 0.080%*** *0.000%*

**Triploid 5.0% 0.02%//** 0.00% **0.03%// 0.40%** 0.000%

**Tetraploid 2.8% 0.02%// 1.20% 0.005%// 0.08%** 0.000%

**Trisomy 21 2.3% 0.94%// 6.10% 0.90%// 2.50% 0.120%**

***Complex Abnormalities 2.3%*** *0.00%// 0.00%* *0.00%****// 0.70%*** *0.000%*

**Aneuploid to Tetraploid 2.2%** 0.00%// 0.00% 0.00%**//** 0.00% 0.000%

***Trisomy 22 2.1%*** *0.00%// 0.00%* *0.00%****// 0.08%***  *0.000%*

**16 Additional Trisomies 7.9% 0.23%//** 0.00% **0.08%// 0.70%** 0.000%

***Diploid->Tetraploid 1.4%*** *0.00%* ***// 2.40%*** *0.00%****// 0.04%*** *0.000%*

***Double Aneuploid 1.3%*** *0.00%// 0.00%* *0.00%****// 0.040%*** *0.000%*

**Trisomy 13 1.2% 0.20%// 3.70% 0.18%// 0.40% 0.005%**

**Trisomy 18 1.1% 0.37%// 6.10% 0.29%// 1.20% 0.015%**

***Other Monosomy(i.e.-21,-11) 0.2%*** *0.00%****// 1.20%*** *0.00%****// 0.40%***  *0.000%*

**47,XXY – Klinefelter 0.0% 0.16%//** 0.00% **0.09%// 0.80% 0.110%**

**Triploid to Aneuploid 0.7%** 0.00%// 0.00% 0.00%**//** 0.00% 0.000%

**Tetraploid to Aneuploid 0.8%** 0.00%// 0.00% 0.00%**//** 0.00% 0.000%

**Balanced Translocations 0.8% 0.06%/ 3.70% 0.08%// 0.40% 0.030%**

**Unbalanced Translocations 0.7% 0.41%//** 0.00% **0.07%// 0.30% 0.010%**

***Mosaic 0.7%*** *0.00%// 0.00%* *0.00%****// 0.40%*** *0.000%*

**Deletions 0.5% 0.14%//** 0.00% **0.09%// 0.08% 0.009%**

***Isochromosomes 0.4%*** *0.00%****// 1.20%*** *0.00%****// 0.040%*** *0.000%*

**Marker 0.07% 0.02%//** 0.00% 0.00%**// 0.08% 0.070%**

***Inversions 0.06% 0.06%/ 1.20% 0.10%// 0.40% 0.020%***

**47,XXX 0 .06% 0.10%/** 0.00% **0.06%/ 0.80% 0.120%**

**47,XYY**  0.00% **0.08%/** 0.00% **0.08%/ 0.80% 0.100%**

**---------------------------------------------------------------------------------**

**TOTAL ABNORMAL 50% 3.1%/33% 2.30%//7.20% 0.640%**

**-------------------------------------------------------------------------------**

**(1)Lebo, Akron, Ohio: 2002-2013**

**(2) Lebo et al, San Francisco, 1992.**

**(3) Vogel and Motulsky, Seattle, 1986, pp. 335. [23]**

**TABLE S2B: 26 Abnormal Karyotypic Categories Detected at 54 Sites**

**Listed by Decreasing Severity.**

***(POC + Newborn = population frequency; CVS/Amnio=consented to procedure)***

***All bold results occur in frequencies of at least 1 in 5,000 positive prenatal samples***

***And 1 in 20,000 positive newborns.***

***54 Sites* detect all aneuploid loci.** (Lebo et al., 2013)

**Sample Source: Products(1) CVS(2/1) Amniocentesis(2/1) Newborns(3)**

**of Conception 1987-95 SF/ 1970-95 SF/ 1986**

**2002-2013 OH 2007-13 OH 2002-2013 OH Seattle**

**------------------------------------------------------------------------------**

**% ABNORMAL 50% 3.1%// 2.3%// 0.6%**

**33.0% 7.2%**

**------------------------------------------------------------------------------**

**TOTAL TESTED: 1449 (1) 5,134 (2)// 21,288 (2)// 54,749(3)**

**82 (1) 2,598 (1)**

**-------------------------------------------------------------------------------**

**Triploid to Aneuploid 0.7%** 0.00%//0.00% 0.00%**//**0.00% 0.000%

**Tetraploid to Aneuploid 0.8%** 0.00%//0.00% 0.00%**//**0.00% 0.000%

**Aneuploid to Tetraploid 2.2%** 0.00%//0.00% 0.00%**//**0.00% 0.000%

**Double Aneuploid 1.3%** 0.00%//0.00% 0.00%**//0.040%** 0.000%

**Isochromosomes 0.4%** 0.00%**//1.20%** 0.00%**//0.040%** 0.000%

***Complex Abnormalities 2.3%*** *0.00%//0.00%* *0.00%****//0.70%*** *0.000%*

***Diploid->Tetraploid 1.4%*** *0.00%* ***/2.40%*** *0.00%//****0.04%***  *0.000%*

**Trisomy 16 6.2%** 0.00%//0.00% 0.00%**//0.080%** 0.000%

**Trisomy 22 2.1%** 0.00%//0.00% 0.00%**//0.08%**  0.000%

**Other Monosomy(i.e.-21,-11) 0.2%** 0.00%**// 1.20%** 0.00%**//0.40%**  0.000%

**Mosaic 0.7%** 0.00%//0.00% 0.00%**//0.40%** 0.000%

**Other Trisomies (not 1,5,19) 7.9% 0.23%//**0.00% **0.08%//0.70%** 0.000%

**Triploid 5.0% 0.02%//**0.00% **0.03%//0.40%** 0.000%

***Tetraploid 2.8% 0.02%//1.20% 0.005%/0.08%*** *0.000%*

**Trisomy 13 1.2% 0.20%//3.70% 0.18%//0.40% 0.005%**

**Trisomy 18 1.1% 0.37%//6.10% 0.29%//1.20% 0.015%**

**45,X – Turner 7.9% 0.29%//4.90% 0.18%//0.40% 0.040%**

**Trisomy 21 2.3% 0.94%//6.10% 0.90%//2.50% 0.120%**

**47,XXY – Klinefelter 0.0% 0.16%//**0.00% **0.09%//0.80% 0.110%**

**Deletions 0.5% 0.14%/**0.00% **0.09%/0.08% 0.009%**

**Unbalanced Translocations 0.7% 0.41%/**0.00% **0.07%//0.30% 0.010%**

**Marker 0.07% 0.02%//**0.00% 0.00%**//0.08% 0.070%**

***Balanced Translocations 0.8% 0.06%/3.70% 0.08%//0.40% 0.030%***

***Inversions 0.06% 0.06%/1.20% 0.10%//0.40% 0.020%***

**47,XYY**  0.00% **0.08%/**0.00% **0.08%/0.80% 0.100%**

**47,XXX 0 .06% 0.10%/**0.00% **0.06%/0.80% 0.120%**

**______________________________________________________________________________**

**TOTAL ABNORMAL 50% 3.1%/33% 2.30%//7.20% 0.640%**

**----------------------------------------------------------------**

**(1)Lebo, Akron, Ohio: 2002-2013**

**(2) Lebo et al, San Francisco,1992. [22]**

**(3) Vogel and Motulsky, Seattle, 1986, pp. 335.**

***Italicized abnormalities not currently validated by microarrays.***
