# Supplementary material for: Analyzing the most frequent disease loci in targeted patient categories optimizes disease gene identification and test accuracy worldwide
Source: J Transl Med. 2015 Jan 21;13:16. doi: 10.1186/s12967-014-0333-8 (PMC4312458; doi:10.1186/s12967-014-0333-8)
Supplement: Additional file 3: Table S3. — Common submicroscopic aneuploid locI by chromosome location and category: [24,48]. [file 12967_2014_333_MOESM3_ESM.doc]

|  | |  | **Additional file 3: Table S3A:**  **Common Submicroscopic Aneuploid LocI.** |  |  |
| --- | --- | --- | --- | --- | --- |
|  | |  | **(All Affected Patient Frequencies;**  **Syndromes with population frequencies)***** |  |  |
|  | |  | **Heterozygous dominant** | **Affected** | **(2pq + q2)***** |
| 157 | | 1 | Trisomy 21 |  |  |
| 158ID | | 2* | **16p11.2 Deletion Syndrome - Autism/ID/Schizophrenia** | 1/115***(T)*** |  |
| 159ID | | 3* | **16p11.2 Duplication - Autism** | 1/127***(T);*** 1/235(K); | 1/3,300 |
| 160 | | 4 | 22q11.21 Duplication Syndrome - DiGeorge Region/Variable | 1/127***(T);*** 1/404(K); |  |
| 161 *S* | | 5 | *7q11.2 Deletion - Williams Syndrome***** | 1/158***(T);*** 1/350(K) | 1/700***** |
| 162 *S* | | 6* | *22q11.21 Deletion Syndrome - DiGeorge/VCFS /Schizophrenia* | 1/211 ***(T)****;* 1/463(K) | 1/7,500 |
| 163ID | | 7* | **1q21.1 Deletion-ID/microcephaly/schizophrenia/cardiac -var** | 1/253***(T)****;* 1/169(K) | 1/5,200* |
| 164 *S* | | 8 | *17p13.3 Miller-Dieker Syndrome* | 1/253***(T)****;* 1/286(K) |  |
| 165ID | | 9 | **1p36.32p36.33 Deletion Syndrome/Facies/Intell/Speech** | 1/316***(T)*** | 1/17,544**** |
| 166ID | | 10* | **16p13.11 Duplication - Variable** | 1/316***(T)*** | 1/7,500**(S) |
| 167 | | 11 | 16p13.11 Duplication - variable | 1/316***(T);*** 1/350(K); |  |
| 168 *S* | | 12* | *15q11.2->q13 Angelman Syndrome - most de novo* | 1/316***(T);*** 1/350(K) |  |
| 169 *S* | | 13* | *15q11.2->q13.1 Prader-Willi Syndrome - most de novo* | ~1/316***(T)****;* ~1/192(K) | 1/16,000* |
| 170 *S* | | 14 | *4p16.3 Wolf-Hirschhorn Syndrome - deletion in 4p16.3* | ~1/316***(T)****;* ~1/192(K); | 1/20,000 |
| 171ID | | 15 | **18p11.32 Deletion - Autism in Safari** | 1/422 ***(T)*** | 1/50,000**** |
| 172ID | | 16* | **8p23.1p23.3 Deletion - mild ID/small/poor growth/cardiac** | 1/422***(T)*** |  |
| 173ID | | 17* | **15q11.2 Duplication - Autism** | 1/422***(T)****;* 1/1575(K) |  |
| 174ID | | 18* | **15q13.2q13.3 Deletion = ID+Epilepsy/Schizophrenia** | 1/422***(T);*** 1/450(K) |  |
| 175 | | 19 | Xp22.31 Duplication -STS | 1/422***(T);*** 1/342(K) |  |
| 176ID | | 9 | **7p22.2p22.3 Deletion - Autism Database** | 1/422***(T)*** |  |
| 177ID | | 21 | **7p22.2p22.3-Duplication - Autism Database** | 1/422***(T)*** |  |
| 178 | | 22 | Iso(18)(p10) | 1/422***(T)*** |  |
| 179 | | 23 | Trisomy 18 | 1/633***(T)*** |  |
| 180 *S* | | 24* | *5q35.3 Sotos Syndrome* | 1/633***(T)*** |  |
| 181 | | 25 | Xp22.31 Duplication - KAL1 | 1/633 ***(T)****;* 1/1969(K) | 1/14,000* |
| 182 *S* | | 26 | *Xp22.31 Steroid sulfatase deficiency* | 1/633***(T)*** |  |
| 183ID | | 27* | **1q21.1 Duplication Syndrome- ID/Autism** | 1/633***(T)*** | 1/16,000**** |
| 184ID | | 28 | **8p21.1 Duplication - Developmental Delay** | 1/633***(T)****;* 1/562(K) |  |
| 185ID | | 29* | **15q13 Duplication - Schizophrenia/Autism** | 1/633***(T)*** |  |
| 186 | | 30 | Xq28q28 -MECP2 Duplication | 1/633***(T);*** 1/1125(K); |  |
| 187 *S* | | 31 | *17p11.2 Smith-Magenis Syndrome* | 1/633***(T)*** |  |
| 188ID | | 32 | **8q21.32 - Deletion Schizophrenia** | 1/1265***(T)****;* 1/984(K) | 1/15,000**** |
| 189 | | 33 | 7q21.3 (SHFM1) Deletion - split hand/foot | 1/1265***(T)*** |  |
| 190 | | 34 | 8p23.3p23.1-Duplication-Variable phenotype (Wheeler, P) | 1/1265***(T)*** |  |
| 191ID | | 35* | **17q12 Deletion - Autism/Schizo/Renal Cysts/Diabetes** | 1/1265***(T)****;*1/2625(K) |  |
| 192 | | 36 | 17p12 Duplication - Charcot-Marie-Tooth Type 1A | 1/1265***(T);*** 1/875(K); | 1/3,000 |
| 193ID | | 37 | **18q21.32q23 Deletion - Schizophrenia** | 1/1265***(T)*** |  |
| 194ID | | 38 | **4q21.2-q24 Deletion - ID/microcephaly/growh rest.: 9pts(Bo)** | 1/1265***(T)*** |  |
| 195 | | 39 | Xq13.2 Duplication - Variable | 1/1265***(T)*** |  |
| 196 *S* | | 40 | *1q41 Deletion - Usher 2A Syndrome -* | 1/1265***(T)*** |  |
| 197 *S* | | 41 | *5p15.2 Cri-du-chat Syndrome* | 0(T) | 1/6,000 |
| 198 *S* | | 42* | *17q21.31 Deletion Syndrome* | 0(T); 1/716(K) | 1/35,000 |
| 199 *S* | | 43* | 1q21.1 TAR (Thrombocytopenia Absent Radium Syndrome) | 0(T); 1/716(K) | 1/16,000 |
| 200ID | | 44 | **2p16.3 Deletion - Intell/Autism/Siezure (Schaaf et al, 2012)** | 0(T); 1/926(K) | 1/150,000**** |
| 201ID | | 45* | **3q29 Deletion Syndrome- Schizophrenia** | 0(T); 1/403 (S) |  |
| 202ID | | 46* | **5q35.3 -Duplication Short Stature/Microceph/speech delay** | 0(T); 1/1750(K) |  |
| 203ID | | 47* | **7q11.2 Duplication - Autism** | 0(T); 1/7875(K) |  |
| 204ID | | 48* | **16p13.11 Deletion Autism/ID/Schizophrenia** | 0(T); 1/984(K) |  |
|  |  |  | **TOTAL** | 121/1265***(T) (9.6%)*** |  |

**Table S3B:**

|  |  | **Common Submicroscopic Aneuploid Loci**  **by Chromosome Location and Category: [50]** |  |  |
| --- | --- | --- | --- | --- |
|  |  | **(All with Affected Patient Frequencies;**  **Syndromes with population frequencies)***** |  |  |
|  |  | **Affected are Heterozygous** | Affected |  |
|  |  | **Mutations are dominant** | Patients | **(2pq + q2)** |
|  |  |  |  |  |
| 157 | 1* | 1q21.1 TAR (Thrombocytopenia Absent Radium Syndrome) | 1/926(K) | 1/100,000-1/200,000**** |
| 158 | 2 | 1q41 Deletion - Usher 2A Syndrome - | 0(T) | 1/6,000 |
| 159 | 3 | 4p16.3 Wolf-Hirschhorn Syndrome - deletion in 4p16.3 | 1/422 ***(T)*** | 1/50,000**** |
| 160 | 4 | 5p15.2 Cri-du-chat Syndrome | 0(T) | 1/20,000-1/50,000 |
| 161 | 5* | 5q35.3 Sotos Syndrome | 1/632 ***(T)****;* 1/1969(K) | 1/14,000* |
| 162 | 6 | 7q11.2 Deletion - Williams Syndrome**** | 1/211 ***(T)****;* 1/463(K) | 1/7,500 |
| 163 | 7* | 15q11.2->q13 Angelman Syndrome - most de novo | ~1/362***(T)****;* ~1/192(K) | 1/12,000-1/20,000* |
| 164 | 8* | 15q11.2->q13.1 Prader-Willi Syndrome - most de novo | ~1/362***(T)****;* ~1/192(K); | 1/10,000-1/30,000 |
| 165 | 9 | 17p13.3 Miller-Dieker Syndrome | 1/316***(T)*** | 1/25,000-1/85,470**** |
| 166 | 10* | 17p11.2 Smith-Magenis Syndrome | 1/1265***(T)****;* 1/984(K) | 1/15,000**** |
| 167 | 11* | 17q21.31 Deletion Syndrome | 0(T); 1/716(K) | 1/16,000 |
| 168 | 12* | 22q11.21 Deletion Syndrome - DiGeorge/VCFS /Schizophrenia | 1/253***(T)****;* 1/169(K) | 1/4,000-1/6,395* |
| 169 | 13 | Xp22.31 Steroid sulfatase deficiency | 1/633***(T)*** | 1/16,000**** |
|  |  | **TOTAL** | **~1/21** | **1/1097** |
|  |  | **ALTERED INTELLECTUAL DEVELOPMENT:** |  |  |
| 170 | 1 | **1p36.32p36.33 Deletion Syndrome/Facies/Intell/Speech** | 1/316***(T)*** | 1/5,000-1/10,000**(S) |
| 171 | 2* | **1q21.1 Deletion-ID/microcephaly/schizophrenia/cardiac -var** | 1/253***(T)****;* 1/286(K) |  |
| 172 | 3* | **1q21.1 Duplication Syndrome- ID/Autism** | 1/633***(T)****;* 1/562(K) |  |
| 173 | 4 | **2p16.3 Deletion - Intell/Autism/Siezure (Schaaf et al, 2012)** | 0(T); 1/403 (S) |  |
| 174 | 5* | **3q29 Deletion Syndrome- Schizophrenia** | 0(T); 1/1750(K) |  |
| 175 | 6 | **4q21.2-q24 Deletion - ID/microcephaly/growh rest.: 9pts(Bo)** | 1/1265***(T)*** |  |
| 176 | 7* | **5q35.3 -Duplication Short Stature/Microceph/speech delay** | 0(T); 1/7875(K) |  |
| 177 | 8 | **7p22.2p22.3 Deletion - Autism Database** | 1/450***(T)*** |  |
| 178 | 9 | **7p22.2p22.3-Duplication - Autism Database** | 1/450***(T)*** |  |
| 179 | 10* | **7q11.2 Duplication - Autism** | 0(T); 1/984(K) |  |
| 180 | 11* | **8p23.1p23.3 Deletion - mild ID/small/poor growth/cardiac** | 1/422***(T)****;* 1/1575(K) |  |
| 181 | 12 | **8p21.1 Duplication - Developmental Delay** | 1/633***(T)*** |  |
| 182 | 13 | **8q21.32 - Deletion Schizophrenia** | 1/1265***(T)*** |  |
| 183 | 14* | **15q11.2 Duplication - Autism** | 1/422***(T);*** 1/450(K) |  |
| 184 | 15* | **15q13.2q13.3 Deletion = ID+Epilepsy/Schizophrenia** | 1/422***(T);*** 1/342(K) |  |
| 185 | 16* | **15q13 Duplication - Schizophrenia/Autism** | 1/633***(T);*** 1/1125(K); |  |
| 186 | 17* | **16p13.11 Deletion Autism/ID/Schizophrenia** | 0*(T);* 1/716(K) |  |
| 187 | 18* | **16p13.11 Duplication - Variable** | 1/316***(T);*** 1/350(K); |  |
| 188 | 19* | **16p11.2 Deletion Syndrome - Autism/ID/Schizophrenia** | 1/127***(T);*** 1/235(K); | 1/3,300 |
| 189 | 20* | **16p11.2 Duplication - Autism** | 1/127***(T);*** 1/404(K); |  |
| 190 | 21* | **17q12 Deletion - Autism/Schizo/Renal Cysts/Diabetes** | 1/1265***(T);*** 1/875(K); | 1/3,000 |
| 191 | 22 | **18p11.32 Deletion - Autism in Safari** | 1/422***(T)*** |  |
| 192 | 23 | **18q21.32q23 Deletion - Schizophrenia** | 1/1265***(T)*** |  |
| 193 | 1 | 7q21.3 (SHFM1) Deletion - split hand/foot | 1/1265***(T)*** |  |
| 194 | 2 | 8p23.3p23.1-Duplication-Variable phenotype (Wheeler, P) | 1/1265***(T)****;* 1/2625(K) |  |
| 195 | 3 | 16p13.11 Duplication - variable | 1/316***(T);*** 1/350(K) |  |
| 196 | 4 | 17p12 Duplication - Charcot-Marie-Tooth Type 1A | 1/1265***(T)*** |  |
| 197 | 5 | Iso(18)(p10) | 1/632***(T)*** |  |
| 198 | 6 | Trisomy 18 | 1/632***(T)*** |  |
| 199 | 7 | Trisomy 21 | 1/115***(T)*** |  |
| 200 | 8 | 22q11.21 Duplication Syndrome - DiGeorge Region/Variable | 1/158***(T);*** 1/350(K) | 1/700***** |
| 201 | 9 | Xp22.31 Duplication - KAL1 | 1/633***(T)*** |  |
| 202 | 10 | Xp22.31 Duplication -STS | 1/422***(T)*** |  |
| 203 | 11 | Xq13.2 Duplication - Variable | 1/1265***(T)*** |  |
| 204 | 12 | Xq28q28 -MECP2 Duplication | 1/633***(T)*** |  |
|  |  | **TOTAL** | 121/1265***(T)***  ***(9.6%)*** |  |

**(T)** Calculated from our 1265 microarray tests.

(K) Kaminsky et al., 2011,**[21]**. Listed ratio calculated from 15,749 cases of intellectual and developmental disabilities.

Table S3 Legend.

Available microarray platforms quantify tens of thousands of sites and delineated all the listed abnormalities with 44,000 sites tested. Microarrays that also quantify polymorphic sites can characterize 92,XXXX, 92,XXYY, and 69XXX cells that arise from three or four gametes but not two. Microarrays also precisely delineate unbalanced translocation breakpoints which may be ordered in place of karyotyping or as a follow up to the unbalanced chromosomal rearrangements. Including male control DNA allows one to quantify the number of Y chromosomal sites. [Additional file 2: Table S2A,B, not italicized]
