# Supplementary material for: Analyzing the most frequent disease loci in targeted patient categories optimizes disease gene identification and test accuracy worldwide
Source: J Transl Med. 2015 Jan 21;13:16. doi: 10.1186/s12967-014-0333-8 (PMC4312458; doi:10.1186/s12967-014-0333-8)
Supplement: Additional file 4: Table S4. — Current platform detection capability [49–56]. [file 12967_2014_333_MOESM4_ESM.doc]

*Additional file 4: Table S4.* ***Current Platform Detection Capability***

***Microarray Rapid DNA Sequencer***

***Quantification SNP Typical Quantification SNP***

***Ratios No SNP Analysis Ratios***

***1. Nearly All Tetraploid Genomes Derived from Diploid Genomes Including Mosaics:***

***Note: All Tetraploid Identified by Karyotyping and FISH.***

*46,XX/92,XXXX No No No No*

*46,XY/92XXYY No No No No*

*92,XXXX No No No No*

*92XXYY No No No No*

***2A. Tetraploid from 4 Gametes: (Rare)***

***Note: All Tetraploid Identified by Karyotyping and FISH.***

*92,XXXY Yes (a) Yes (c) Yes (b) Yes (b)*

*92,XYYY Yes (a) Yes (c) Yes (b) Yes (b)*

*92,XXXX No Yes(d) No Yes(d)*

*92,XXYY No Yes (d) No Yes(d)*

***2B. Triploid from 3 Gametes. (Nearly All Partial Moles derived from 2 Sperm-Diandric):***

***Note: All Triploid Identified by Karyotyping and FISH.***

*69,XXX No Yes (c) No Yes (b)*

*69,XXY Yes (a) Yes (c) Yes (b) Yes (b)*

*69.XYY Yes (a) Yes (c) Yes (b) Yes (b)*

***2C. Complete Diploid Moles from 2 Sperm:***

***Note: Detected by Abnormal Fetal Chorionic Villus Morphology,***

***Increased maternal risk for choriocarcinoma.***

***Not Detected by Karyotyping or FISH.***

*46,XY No Yes/LOH No Yes/LOH*

*46,XX No Yes/LOH No Yes/LOH*

***3. Balanced Rearrangements:***

***Note: Microscopically Visible Identified by Karyotyping and FISH.***

*Translocations No Being validated No Being validated*

*Inversions* *No Being validated No Being validated*

***__________________________________________________________***

***a.*** *Enabled by X and Y sex chromosome quantification including Y chromosome containing control.*

***b.*** *Enabled by autosome and sex chromosome polymorphic quantification.*

***c.*** *Enabled by autosome and sex chromosome quantification with Y chromosome containing control.*

*d.Enabled by polymorphic quantification.*

***Table 6. Legend:***

***1. Tetraploid from diploid:***

*Typically a tetraploid cell arises from a normal diploid 46,XX or 46,XY cell with AA, AB, or BB single nucleotide polymorphic sites. This diploid cell fails to divide resulting in a tetraploid cell with a 92,XXXX or 92,XXYY karyotype and AAAA, AABB, or BBBB single nucleotide polymorphic sites. All tetraploid results explain fetal demise. [49,50,51] FISH and karyotyping readily distinguish these categories in single cells by analyzing intact nuclei or associated metaphase chromosomes. A mosaic tetraploid cell line or conceptus with diploid and tetraploid karyotypes would result in the same polymorphic ratios for both cell types as the diploid progenitor cell. Thus tetraploid cells could not be distinguished from normal diploid progenitor cell results by quantifying genomic sites with microarrays or by sequencing to analyze total dissolved DNAs because the total number of DNA targets per cell all doubled during genomewide tetraploidization to maintain the relative ratios of any genomewide targets. The difference in the number of DNA sequences per cell would not be discernible because the cell nuclei have been disrupted during DNA extraction. Our cytogenetics laboratory reports mosaic tetraploid karyotypes when the proportion of tetraploid nuclei comprise at least 20% of the total. (Table 2B) Rare exceptions result from three paternal gametes and one maternal gamete.[51]*

***2. Hydatidiform Mole Summary****.*

*A “Hydatidiform mole (HM) is a pregnancy with no embryo but with degeneration of chorionic villi. Moles occur in ~1 in every 1500 pregnancies in Europe and North America” [52] and up to 10-fold more frequently in other populations. “Recurrent hydatidiform moles in a single-family member occur in 0.6–2.57% of all molar cases and in rare cases in at least two related women. The majority of patients present with vaginal bleeding or are discovered during ultrasonography. After diagnosis, moles are evacuated by dilatation and suction curettage and the patients followed up with a series of serum hCG estimations until the hCG level falls to a non-pregnant state. Up to 15% of cases have remaining molar tissues that invade the uterine wall leading to an invasive mole or in rare cases (2–5%) to a choriocarcinoma, a malignant, rapidly growing, and metastatic cancer.”[52]*

*HMs are divided into two types: complete hydatidiform moles (CHMs) and partial hydatidiform moles (PHMs). CHMs are characterized by hydropic degeneration of all villi and absence of embryo, cord, and amniotic membranes. In CHMs, all the villi are enlarged with cisternae, avascular, and surrounded by excessive trophoblastic proliferation. PHMs are characterized by focal trophoblastic proliferation with a mixture of normal-sized villi and edematous villi. The trophoblastic proliferation is less pronounced than in complete moles. An embryo, cord, and amniotic membranes are usually present in partial moles. The most recent reports estimate that 80% of CHMs have a diploid genome and are androgenetic: 60% are monospermic and 20% dispermic . The remaining 20% have a biparental genome.”[52]*

*In our laboratory, review of POC villi by a Pathologist reveals hydatidiform mole morphology which is confirmed by immunohistochemical testing of p57(KIP2) gene expression and karyotyping to determine whether maternal expression of this paternally inactivated imprinted gene is noted in syncytiotrophoblasts of early hydatidiform moles.[53,54] Subsequent monitoring assures the mother does not develop choriocarcinoma from any remaining molar tissue. Although typical partial moles with 69 chromosomes with two diandric haploid genomes and rare partial moles with 92 chromosomes and three diandric haploid genomes [Table Section 4A, above] are at lower risk of developing into a choriocarcinoma, karyotypes are used to confirm ploidy. Alternatively, polymorphic microarrays or rapid sequencing and analysis of polymorphic sites could define these genomes unambiguously.*

***2A.*** ***Tetraploid Moles from 4 Gametes****.*

*Tetraploidy in very unusual reported cases resulted following fertilization of a single ovum by three sperm.[56] These cases can be identified by platforms that quantify polymorphic sites with AAAA. AAAB. AABB, ABBB, and BBBB polymorphic sites with all four haplotypes contributing to these ratios. Karyotyping and FISH distinguish tetraploidy directly but do not distinguish the number of gametes that gave rise to the tetraploid chromosome number. Follow up analysis of beta-HCG can identify whether this category exists and needs to be followed. Current polymorphic microarrays could identify 92,XXXX or 92,XXYY tetraploid cells that arose from four gametes at fertilization.*

***2B. Partial Moles:***

***1.*** ***Triploid Moles from 3 gametes.***

*Nearly all triploid conceptuses arise from fertilization of an egg by two sperm (diandric) tetraploid conceptuses [35% of total; Ref.55]. These are defined by karyotyping and rapid FISH. These are also characterized readily by microarrays that record the relative numbers of single polymorphic sites to distinguish the AAA, AAB, ABB, and BBB genotypes. Earlier microarrays that only quantified total genomic sites compared to all sites could not identify 69,XXX triploid cells. Sequencers that record the relative number of each nucleotide location among the total also reveal these results.*

***2C. Complete Moles: Diploid from 2 male gametes.***

*Pathologists identify early hydatidiform moles by gross villous enlargement in submitted POCs [55] and follow this observation with immunohistochemical testing of p57(KIP2) gene expression to determine whether maternal expression of this imprinted gene is observed in syncytiotrophoblasts.[53,54] The diploid chromosome number of complete moles is confirmed by karyotyping or FISH. Because the X chromosomal genes are required for survival, complete moles with two diandric haplotypes always have 46,XX or 46,XY karyotypes which may arise from 1 or 2 sperm. Quantifying microarrays will reveal substantial genomewide loss of heterozygosity (LOH) at polymorphic sites with AA or BB results without AB sites for extensive stretches because the gametes are only derived from the father. Ordered testing in any patient is based upon availability within available resources.*

***3. Balanced Rearrangements:***

*Rearrangement at two or more locations may result in balanced translocations and inversions. Reciprocal microscopically visible balanced translocations and inversions are readily characterized by a few consistent karyotypes with banded chromosome locations. These comprise 1.72% of abnormalities in products of conception (POCs). Previously platforms have been developed to identify frequent gene translocations like acute lymphoblastic leukemia.[56] Nevertheless, a single reciprocal translocation with 6 basepairs deleted from two unique chromosome regions was identified by genomewide sequencing fetal DNA among total DNA in maternal circulation.[17]*

***Genomewide Platforms Being validated:***

*Genomewide analysis to detect* *de novo balanced rearrangement at any location would require analysis of unique identifiable sufficiently long chromosome regions analyzed together to identify the origin of both fused chromosome regions from at least two locations in a single location.[51] Given the ~2,370,000 listed copy number variants (Toronto Database) of all sizes from a few basepairs to millions of basepairs and the propensity of recombinations in similar sequences [i.e. ref.36], the proportion of informative translocations should be determined by karyotyping and/or segregating polymorphisms in relatives could be tested readily by targeted molecular analysis. Balanced translocations between chromosome centromeres and/or involving other repetitive chromosome heteromorphisms including 1qH, 9qH, and 16qH are likely to be the overlooked given the regions of repetitive sequences on both sides of the rearrangement. Although these regions are not considered to contain phenotype altering gene sequences, subsequent meiotic rearrangements can readily result in decreased fertility and substantially increased frequencies of abnormal conceptuses. Nevertheless, a balanced fetal translocation was identified from circulating fetal DNA in maternal plasma.[17] At the same time, extensive genomewide microarrays and focused analysis would have to be used for this currently reported application. Genomewide high density microarrays can be used immediately to follow up the apparently balanced karyotyped translocations in CVS and amniocyte samples for submicroscopic deletions and duplications. In contrast, karyotyping 3 cells would readily identify microscopically visible balanced translocations. Targeted microarrays would be used for simpler clinical applications.*
